# *UltraStat*: Ultrafast Spectroscopy beyond the Fourier Limit Using Bayesian Inference

**DOI:** 10.1021/acs.jpca.4c04385

**Published:** 2024-10-16

**Authors:** Elad Harel

**Affiliations:** Department of Chemistry, Michigan State University, 578 South Shaw Lane, East Lansing, Michigan 48824, United States

## Abstract

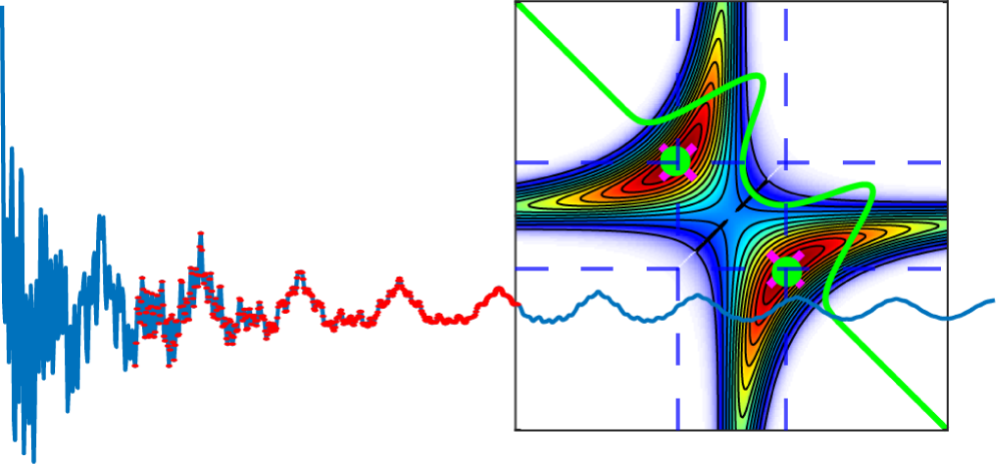

The discrete Fourier transform (dFT) plays a central role in many ultrafast experiments, allowing the recovery of spectroscopic observables from time-domain measurements. In resonant experiments when population relaxation and coherence components of the signal coexist, the dFT is usually preceded by multiexponential fitting to remove the large population term. However, this procedure results in errors in both the recovered decay rates and the line shapes of the coherence spectral components. While other methods such as linear prediction singular value decomposition fit both terms simultaneously, they are limited to specific models that may not represent the true signal. These methods do not allow for systematic noise analysis or error estimation and require *a priori* knowledge of the signal rank. Here, we describe a general approach to parameter estimation in ultrafast spectroscopy—*UltraStat*—grounded in Bayesian analysis without the limitations set by Fourier theory. Using simulated, but realistic data, we demonstrate in a statistical sense how *UltraStat* provides accurate parameter estimation in the presence of many experimental constraints: noise, signal truncation, limited photon budget, and nonuniform sampling. *UltraStat* provides superior resolution compared to the dFT, up to an order of magnitude in cases where the line shapes are well-approximated. In these cases, we establish that primarily noise, not sampling, limits spectral resolution. Moreover, we show that subsampling may reduce the number of acquired points by 90% compared to the Nyquist–Shannon criteria. *UltraStat* greatly improves parameter estimation by providing statistically bound spectral and dynamics analysis, pushing the limits of ultrafast science.

## Introduction

Since the invention in the 1960s of mode-locked lasers, which generated ultrashort pulses of light on the order of femtoseconds (10^–15^ seconds), ultrafast science has experienced exponential growth across many subfields of physics, chemistry, biology, and materials science. Some of the most compelling examples include capturing the breaking and forming of chemical bonds,^1^ the movement of energy in proteins,^1,2^ and the transfer of electrons across material interfaces.^3^ Yet, for all the advancements in the field from next generation laser sources in the THz,^4^ IR,^5^ visible,^6,7^ UV,^8^ and X-ray,^9^ a multitude of novel nonlinear techniques,^10−13^ ultrasensitive detection methods down to single molecules,^14,15^ and the application of these methods to increasingly complex systems,^16−18^ the analysis tools have remained mostly unchanged.^11,19−21^

With notable exceptions,^19,20^ oftentimes the signals arising from time-domain ultrafast experiments are analyzed using multiexponential fitting (MEF) and Fourier analysis (via the dFT). Both methods are attractive for their simplicity and speed. In the presence of resonant excitation, the system is promoted into an excited state whose relaxation dynamics is often described by a weighted sum of exponential decays. When the excitation source is broad, multiple excited states may be accessible, each decaying at a different rate according to the kinetic rate equations. Therefore, MEF appears to be, at least on the surface, an appropriate means to extract the population decay rates in the presence of noise. However, such’population’ states are not the only states excited by ultrashort pulses according to limits set by their time-bandwidth product. Coherence, or superposition states, nearly always coexist, especially in condensed phase systems that have a high density of states.^13^ Recent theoretical work from our group^22^ has shown that even when the coherence signals are much weaker than the population signals as expected for resonant excitation conditions, the rates extracted by MEF under all noise conditions including zero noise, are inaccurate. This is because the MEF does not apply the correct hypothesis when other signals besides those exhibiting population decay are present.

In many time-domain ultrafast experiments, in addition to extracting the population decay rates, information about the coherence frequencies and dephasing rates is desired.^23,24^ A common practice is to subtract the fit from MEF to obtain the residual signal, which is then Fourier transformed to obtain the spectrum. This seems reasonable as the Fourier transform is the frequency representation of the time-domain signal. However, the dFT (implemented numerically using the FFT algorithm), which is the Fourier transform used on discretely sampled time-domain data, represents the signal as sum of *nondecaying* complex exponential functions. This representation fails to accurately describe the true spectrum when there is noise, the signals decay in time, and the sampling is finite. Clearly, these requirements do not hold in any ultrafast experiment. This fact has been long appreciated in other fields that analyze time-domain signals such as nuclear magnetic resonance (NMR)^25^ and mass spectrometery.^26^ In ultrafast spectroscopy, however, the MEF inadequacies further compound the dFT problem because the residual does not truly represent the coherence-only part of the signal, so the dFT results in distortions of features in the low-frequency part of the spectrum where the exponential terms reside in the frequency domain. The result of combining MEF and the dFT, therefore, is inaccurate parameter estimates for the exponential decay rates, coherence frequencies, amplitudes, and coherence dephasing rates (via the line widths). The magnitude of these deviations from the ground truth (GT) spectroscopic parameters strongly depends on the level of noise, the relative strength of the population and coherence components, the sampling rate and signal bandwidth, and the decay and dephasing rates, among others.

Beyond the MEF/dFT approach, there have been notable examples in the literature where ultrafast experiments are analyzed by more sophisticated approaches that do consider both coherences and populations components to the signal, as well as other signal contributions such as the instrument response function (IRF).^27,28^ One notable method described in detail later is linear prediction singular value decomposition (LPSVD), which specifically accounts for signals that are composed of a sum of complex exponentials functions which accounts for both coherences and population terms that are represented by such functions. Other methods explicitly state the mathematical form of the signal and perform a global fit to all the parameters in the model for analyzing data from spectrally resolved pump–probe spectroscopy and multidimensional coherent spectroscopy and imaging.^29,30^

Here, we describe a more general approach to parameter estimation in ultrafast spectroscopy based on Bayesian inference that we call *UltraStat*, building on our previous work on this topic.^22^ Bayesian analysis offers several important advantages over other methods like maximum likelihood, some of which are exploited here, while others are discussed as starting points in future analysis. These advantages include but are not limited to incorporation of prior information,^30^ full probability distribution for parameters, handling of complex models such as those with latent variables, model comparison and selection, predictive uncertainty, adaptability to new data, and robustness to small sample sizes. While analyzing the influence of all these factors is beyond the scope of this work, here we focus primarily on exploring the influence of noise in the recovered parameters using *UltraStat* compared to other methods including MEF-dFT and LPSVD, and to demonstrate unique features of the method that surpass the Fourier limit under certain conditions.

The paper is organized as follows: The first section provides a detailed theoretical analysis of the *UltraStat* method which combines variable projection and Bayesian inference. Bayesian analysis is then introduced as a general approach to parameter estimation from a probabilistic perspective. We detail the connection between the posterior, likelihood, and prior probabilities within the context of time-domain signals. The theory naturally allows estimation of the parameter errors–a feature not available in the dFT and many other analysis methods. In the next section, we introduce a robust algorithm for determining the parameters even when the number of terms is unknown by exploiting statistical information on the noise which may be either measured independently or estimated from the signal itself. The algorithm takes advantage of the Jacobian and Hessian of the objective function, which may be calculated analytically. Next, we present the results of *UltraStat* on a variety of simulated data, starting from noiseless pump–probe experiments. We then add realistic experimental constraints: noise, data truncation, and nonuniform sampling. *UltraStat* is compared to MEF/dFT and LPSVD. We show that *UltraStat* recovers the parameters accurately even when these other methods fail. A simple example where priors can improve the accuracy of the recovered parameters at high noise levels is also presented. We conclude by discussing the benefits, challenges, and opportunities when applying *UltraStat* to real experimental data.

## Theory

### Variable Projection

We begin by discussing variable projection (VP).^29,31^ The problem we are trying to solve is finding a set of parameters, **Φ**, that best describe some data set, S, given a particular hypothesis. Later, we will discuss hypothesis creation and development. Let us assume that the hypothesis, which is a statement about the mathematical form of the signal with respect to a set of model functions, is known. These functions, which depend nonlinearly on the parameters, **Φ**, describe the data based on some physical knowledge of the signals being measured. A key assumption in VP is the so-called linear mixing model in which the signal may be decomposed into the product of a term, M(**Φ**,t), that describes the model functions (which, depend on the nonlinear parameters) and a term, B(q) that represents the amplitudes,
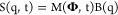
1where we have introduced the variable q to represent one or more experimental dimensions that are not captured directly by the model. For instance, in a coherent multidimensional experiment,^32^ q may represent one or more interpulse delays or a wavelength dimension in spectrally resolved experiments. In an ultrafast imaging experiment,^29,33^ q may represent one or more spatial dimensions, or mixed spatial/spectral/temporal dimensions. Another possibility is to coanalyze experiments under different conditions (e.g., varying the pump or probe wavelength or altering the sample), so that q represents an index corresponding to different data sets. In any of these’multiexperiment’ variations, the goal of the analysis would be to find the parameters, **Φ**, that are common to all the experiments independent of the amplitudes. This could be particularly powerful when coanalyzing thousands of experiments, where fitting the amplitudes directly would be computationally prohibitive. In this paper, we will only treat “q” as a dummy variable to highlight the basic features of *UltraStat*, but we include it for the sake of generality and to emphasize that its inclusion is naturally integrated into the theory.

We may represent S as a matrix of dimensions N_t_ × N_q_. In a one-dimensional spectroscopy experiment as there is no q dependence, we may replace the equation with S(t) = M(**Φ**,t)B, that is, N_q_ = 1. The matrix, M(**Φ**,t) has dimensions N_t_ × M, where M is the number of model functions, and the columns represent the model functions, *M*_*j*_(*t*). We only consider the case where each model function depends only on time, i.e., that the signal is separable into a product of a time component and a spatial component. Finally, B(r), is a matrix of dimensions M × N_q_. Physically, B represents the relative weights of the model functions, i.e., the amplitudes. In an imaging scenario, q corresponds to the pixels so that the rows of B are “hyper-spectral” images, each one corresponding to a single model function.

To fit the data, we aim to find the set of nonlinear parameters, **Φ** that minimize the least-squares residual,
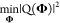
2where
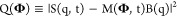
3

As the amplitudes are unknown, we may consider that the terms in B that minimize Q are those that satisfy,
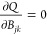
4where j = 1,2, ⋯, M and k = 1,2, ⋯, N_q_. Using this condition, we can write,
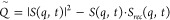
5where
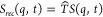
6and
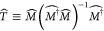
7is an N_t_ × N_t_ matrix (see Supporting Information for derivation). The parameters **Φ** that we seek are those that minimize Q̃/2σ^2^, where σ^2^ is the noise variance. Note that in eq 3, the objective function only depends on the parameters, **Φ** and are independent of the amplitudes. As already mentioned, this greatly simplifies the search problem.

### Bayes Theory

On its own, VP is a powerful method to determine the parameters in a least-squares sense. However, in the presence of noise and when prior information about the parameters is available, it is advantageous to use Bayesian analysis^34^ to achieve greatly improved estimates while providing error bounds. As the objective function in eq 2 is ill-conditioned in the presence of noise, the inclusion of priors may be necessary to obtain good parameter estimates as considered later.

In its simplest form, Bayes theorem states that the joint probability, P(A,B|I) is given by,
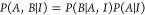
8

In other words, the joint probability of A and B given the information I is equal to the probability of B given A and I multiplied by the probability of A given I. Using the sum and product rules of probability, Bayes theorem may be recast as,
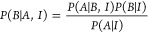
9

In the language of the parameter estimation problem,
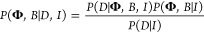
10where D represents the data and I the prior information, and **Φ** and B are the parameters and amplitudes, respectively. The left-hand term is the *posterior probability*, which is the probability of the parameters given the data and prior information. The denominator, p(D|I) is called the *marginal probability* of the data given only the prior information, which we will treat as a constant. The term, p(D|**Φ**,B,I) is called the *likelihood function*, which is the probability of the data being measured given the set of parameters and amplitudes and the prior information. The last term, p(**Φ**,B|I) is the *prior probability*, which is the probability of the parameters given only the prior information. Bayes theorem establishes that the posterior probability, which is a test of the hypothesis vis-a-vis the parameters and amplitudes, may be strengthened by knowledge of the prior. The prior encapsulates knowledge about the physical origin of the signal which could translate to specific bounds on the parameters and amplitudes or other information available from other experiments and theory.^35−37^ In ultrafast spectroscopy, for instance, a prior could be in the form of bounds on the frequencies of the coherences that are limited by the pulse bandwidth. Alternatively, the prior may indicate something about the possible range of rates determined by other experiments or the presence of specific coherence pathways depending on the phase matching geometry^38^ or other signal isolation methods.^39^

To transform Bayes theorem into a practical method, we start with the likelihood function, which may be estimated based on the properties of the noise. For simplicity we assume that the noise is normally distributed with a known standard deviation, σ (i.e., Gaussian noise). Specifically,
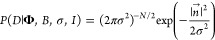
11where  = (n_1_,n_2_,···, n_N_) is the discretely sampled noise vector of the continuous function, n(r,t) = S(r,t) – S_rec_(r,t). Using the definition of Q from the previous section, we find the likelihood function to be,
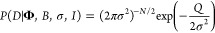
12

Therefore, according to Bayes theorem, the posterior probability is given by,
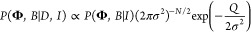
13where the proportionality constant is the marginal probability that we have ignored.

To obtain the posterior probability independent of the amplitudes, we need to integrate this posterior over all the amplitudes,
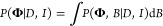
14

Since the integral is highly peaked at the maximum of P(**Φ**,B|D,I), which occurs at the minimum of Q, we may simply substitute for Q the term Q̃ derived earlier. This gives for the posterior probability,
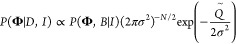
15

Taking the logarithm of both sides and ignoring constants, we find,
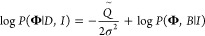
16

This expression for the logarithm of the posterior is the objective function we are trying to maximize as a function of the parameters and amplitudes. However, the prior,  is not expressed in a convenient form because the amplitudes depend on **Φ** according to the VP method prescribed earlier. We may use Bayes theorem again to write,
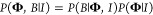
17

Using this expression, we may rewrite the logarithm of the posterior as,
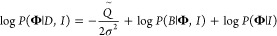
18

If no information about the amplitudes is known, we may ignore the second term on the right-hand side, but for the sake of generality, we will leave this expression as it is. Note that at this point the theory makes no assumptions about which model functions to ascribe to the signal. The optimal parameters are found using an optimization algorithm (see Supporting Information). Further, one can estimate errors for the nonlinear parameters and amplitudes by computing the Hessian of the objective function (see Supporting Information).

## Results

In our previous work on the use of Bayesian inference on ultrafast spectroscopy signals, we developed a simple model consisting of a small number of components. Different scenarios were considered including noiseless signals as well as signals with low- to moderate levels of noise. Here, we introduce signals with significantly more spectral components and explicitly include a phase term for each coherence element. We simulate the signal with different instances of noise to evaluate how spectral recovery is affected by noise in a statistical sense. We first focus on two noiseless models—Model 1: coherence-only, and Model 2: coexistence of coherence and population terms. Then, we add noise to the second of these models, which we call ‘Model 3′. We further consider a simpler two-level system, ‘Model 4′, when testing the resolution limits of *UltraStat*. For the first three models, in addition to analysis by MEF/FFT and *UltraStat*, we apply linear prediction singular value decomposition (LPSVD),^40^ a non-Fourier method that has been used in some previous work.^41,42^ In LPSVD, the signal is decomposed into a sum of complex exponential functions, e^i2πvt+iϕ−Γt^, where *v* is the frequency (units of THz), ϕ is the phase (rad), and Γ is the dephasing (decay) rate (units of THz) for the coherences (populations). LPSVD takes advantage of the fact that the signal is linearly dependent on some integer L of preceding values which are bound according to the number of signal components and measured time points. Using this linear prediction property, it is possible to extract the frequencies, phase, and decay rates exactly in the least-squares sense and in the absence of noise. When noise is present, then an SVD procedure must be used to stabilize the solution as it is otherwise ill-conditioned. The truncated SVD assumes knowledge of the rank or number of components in the signal, which is generally not available *a priori*. In the absence of noise, LPSVD performed at the correct rank and *UltraStat* give the same result to within numerical error, recovering the ground-truth (GT) parameters (the parameters used to generate the model) and the GT spectrum (i.e., the sum of all the individual spectral components) exactly. However, the presence of even a moderate amount of noise leads to substantial errors in the LPSVD algorithm as will be discussed below. The models we consider below consist of a series of modes, each with a Lorentzian line shape. While *UltraStat* is agnostic to the line shape (any model function may be incorporated), this allows us to perform direct comparison to LPSVD which can only consider Lorentzian line shape functions. Further discussions about the line shapes will be presented later.

### Model 1—Coherence-Only, Noiseless

We first consider the ideal case in which there are no population signals and no noise. The signal is generated as the real part of the sum of complex exponential “model” functions of the form, exp(i2πvt + iϕ – Γt). We consider *M* = 8 coherence components.

**Figure 1:**
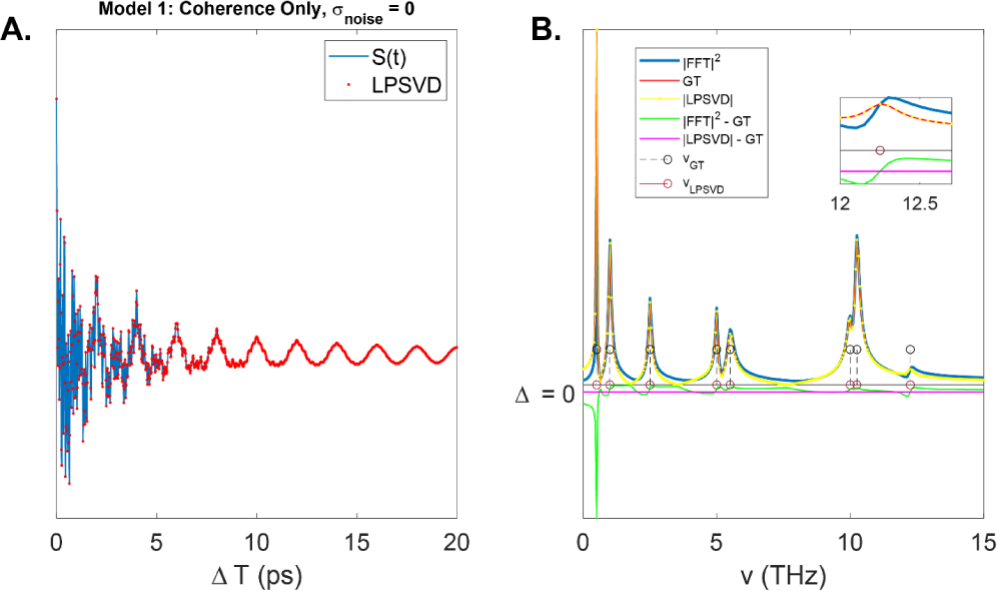
(A) Model 1 composed of only coherence components (blue curve) and LPSVD fit (red dots). In the case of no noise, the LPSVD perfectly recovers the signal when the rank is known. (B) Power spectrum (blue), ground-truth (red), and LPSVD (yellow) spectra. Green curve shows the errors in the FFT power spectrum, while magenta curve is identically zero. The GT frequencies are shown as circles. Inset displays a zoomed in region near 12 THz where the FFT power spectrum appears to show a frequency shift compared to the GT spectrum.

The time-domain signal is shown Figure 1A (blue curve) up to a maximum time delay of 20 ps. The sampling rate is Fs = 1/20 fs so that all the frequencies are well within the Nyquist bandwidth, 2 × max(v)<1/Fs = 50 THz. The LPSVD signal was evaluated at sequentially larger rank values until the residual between the original and recovered signal was zero. This occurs at *m* = 16 because each term in the signal contributes two terms in the complex exponential cos(x) = (e^ix^ + e^–ix^)/2. The FFT power spectrum is shown in blue, while the red and yellow curves show the ground-truth spectrum (GT) and LPSVD recovered spectra, respectively. Again, note that the GT spectrum is the sum over all the individual spectral components, while the GT parameters are those corresponding to the individual components. In any ultrafast experiment, it is the individual spectral components that are ideally recovered. Since the spectral parameters (frequency, dephasing rate, and phase) of each component in the model and LPSVD-recovered signal are known, we can calculate the total spectrum exactly from the sum of the continuous FT of the model functions (decaying sinusoidal form), which are Lorentzian functions. Because we are applying the continuous FT on a noiseless signal of infinite duration, the analytical solution represents the true GT spectrum of the system. Note, the GT and LPSVD spectrum agree within numerical error (difference is shown as the magenta curve) because the LPSVD is the true solution in the least-squares sense in the absence of noise. However, the FFT power spectrum does not agree with the absolute value of the GT/LPSVD solution (difference is shown in the green curve). This is evident at each spectral band but is most pronounced at low frequency (e.g., v_1_ = 0.5 THz, Γ_1_ = 0.05 THz). The reason is that the signal does not fully decay to “zero” within the measured time window, leading to errors in the FFT amplitudes due to truncation. In fact, none of the signal components fully decay to zero within any finite time period, but the amplitude error is most pronounced for those components with line widths narrower than the Fourier limit, δv = 1/*T*_max_. In such cases where the signal does not fully decay, an apodization function such as matched exponential decay function is typically applied to force the signal to go to zero smoothly. The FFT, then, is the convolution of the apodization function spectrum and the original signal spectrum. While this procedure reduces or eliminates truncation artifacts such as sinc lobes that manifest as spectral ringing, it cannot eliminate errors in signal amplitude. Apodization is particularly problematic for improving the signal-to-noise when there are components in the signal that decay at different rates so that no single apodization function can match all components simultaneously. However, it is not only the amplitudes that are inaccurate; the frequency position of the peaks in the FFT power spectrum may also deviate from the nominal value. This is illustrated for the peak at v_6_ = 12.25 THz, whereby the FFT shows the signal maximum at 12.3 THz (Figure 1B, inset). This occurs because the phase of this spectral component is π/2 out of phase with the neighboring components near 10 THz. While not necessarily a large difference, the fact that the relative phase of the spectral components can alter their apparent position in the FFT power spectrum is concerning when small peak shifts are being interpreted as having a physical significance.

### Model 2—Coherences and Populations, Noiseless

**Figure 2:**
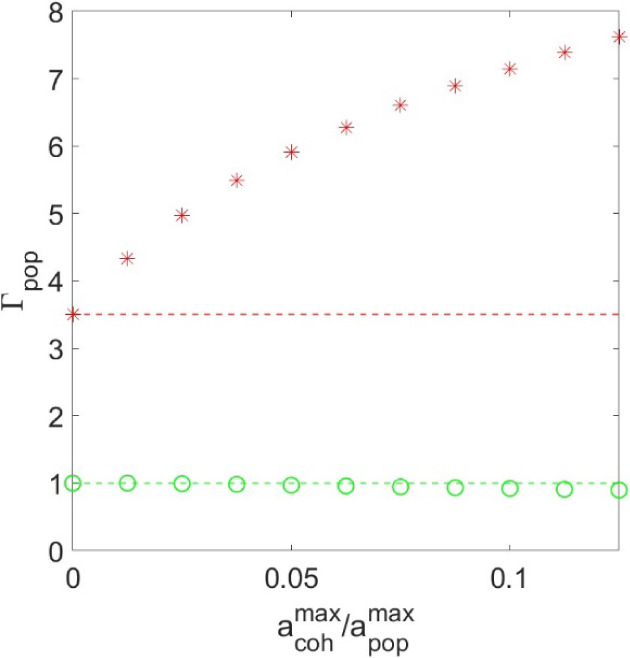
Effect of relative amplitude of coherences to populations on the recovered population decay rates using biexponential fitting.

Next, we consider the same coherence model but with two additional population components added, again without noise. As before, the LPSVD reconstruction reproduces the signal components when the rank is now set to *m* = 18 to account for the two additional population components. Not only are the frequency components identified correctly, but also the decay rates for both population terms, . Note, the amplitude of the population terms are 1–2 orders of magnitude larger than the coherence components in this model. Next, we apply a biexponential fit to the signal, which results in a better fit in the least-squares sense than that for a single exponential. The recovered rate,  deviates from the GT value (3.5 THz) by more than 20%. Changing the relative amplitude of the coherences with respect to the populations has a dramatic effect on the recovered population rates, especially for the fast component as shown in Figure 2.

**Figure 3:**
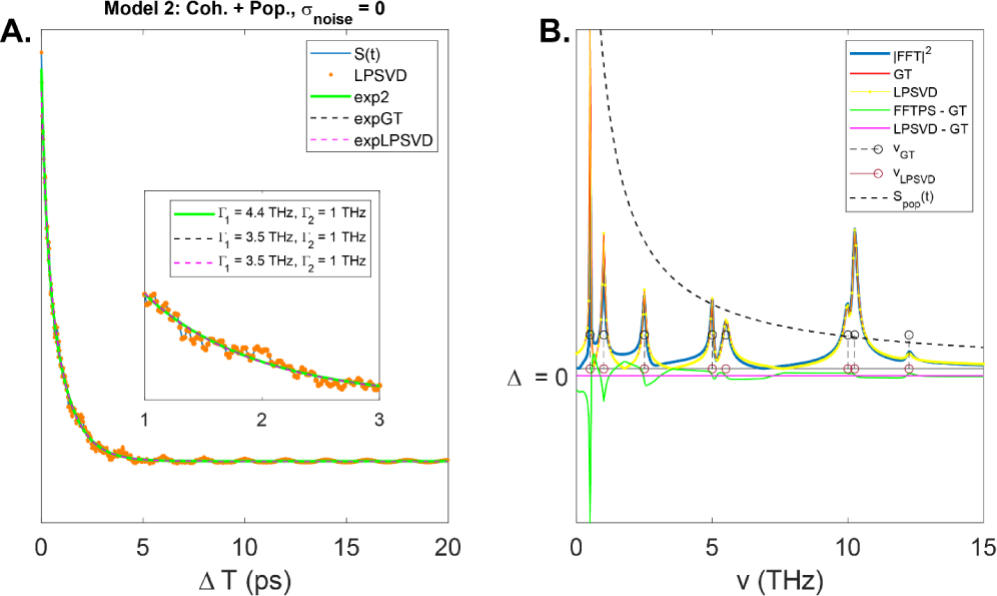
(A.) Model 2 composed of both coherence and population decay components. Biexponential fit (green curve) to the original signal as well as the true population-only signal (black dashed) and recovered LPSVD zero-frequency signal (magenta dashed). The inset shows a zoomed in view in the 1–3 ps range. Note, the recovered biexponential fit is different for the fast component compared to the GT value. (B) Recovered spectra: FFT power spectrum (blue), GT (red), LPSVD (yellow). The spectrum of the population-only signal is overlaid (black dashed) to show which components are most effected in the coherence spectra.

For instance, when the maximum coherence amplitude,  is only 10% of the maximum population amplitude, , the deviation from the GT value for the fast component (dashed red line) is more than 100%. As we highlighted in our previous work, this deviation is not a result of shortcomings in the fitting algorithm, but rather the consequences of fitting the signal to the incorrect model. Subtracting the biexponential fit from the original signal and applying an FFT on the residual results in the power spectrum shown in Figure 3B. As before, we see excellent agreement between the GT and LPSVD, but the FFT deviates even more substantially than in the coherence-only model because of the incorrect rates recovered from MEF. The errors in the low frequency part of the spectrum are more pronounced because this is the region where the residuals of the exponential terms reside (dashed black curve in Figure 3B shows that population-only component of the signal). In conclusion, the presence of the coherences alters the recovered rates, while errors in the rates alter the amplitudes and line shapes of the recovered coherence spectra, even in the absence of noise.

### Model 3—Coherences and Populations, with Noise

The previous two models demonstrate that even in the absence of noise, the dFT provides inaccurate parameter estimation, especially for frequencies and decay/dephasing rates, while it may result in inaccurate frequency estimation for congested features where phase interference is significant. Next, we consider what happens in the presence of a moderate level of noise which is representative of many ultrafast experiments. We only consider Gaussian distributed noise, characterized by a noise variance, σ^2^. We simulated 25 instances of noise, drawn from a Gaussian probability distribution function (pdf) with the same noise variance.

**Figure 4:**
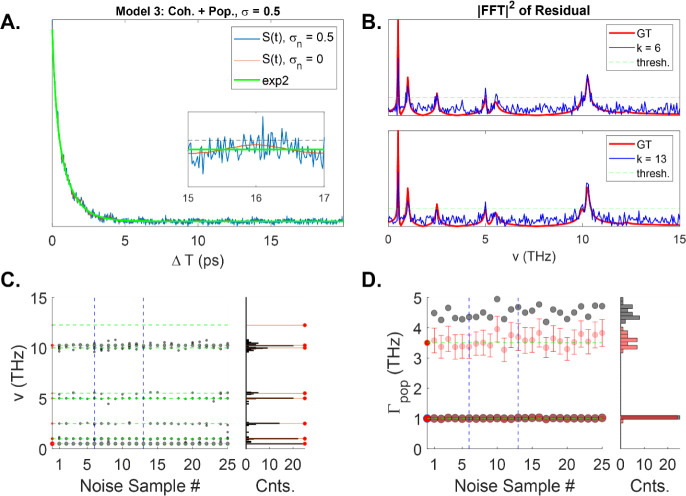
(A) Time-domain trace of model 3. Inset shows that the noiseless signal (red) falls below the noise floor (blue) in less than the full measurement period. (B) FFT power spectrum at two noise instances. Green dashed line shows the threshold value. (C) Analysis of the detected coherences above the threshold value for 25 different noise instances. Right plot shows a histogram of the results compared to the GT frequencies and counts. (D) Analysis of the detected population rates from biexponential fitting. Red circles shows the results of fitting to a population-only signal (errors bars are at the 95% confidence interval). Gray circles show the results from biexponential fitting to the noise signal that includes both populations and coherences. Histogram of both results shown on the right. The recovered rates from MEF are systematically different than the “true” values (red circles) for the fast component.

For one of these instances, the time-domain signal is shown in Figure 4A, along with a biexponential fit to the data (green curve). The inset shows a zoomed in region between 15 and 17 ps, where the noiseless signal (red curve) is superimposed on the noise (blue curve). We display the FFT power spectrum for two noise instances (Figure 4B). An immediate problem that arises when examining these spectra is how to choose which peaks represent real signals and which peaks represent noise. While there may be many ways to formulate criteria, we choose to identify a threshold value based on analyzing the largest value in the noise power spectrum among all the noise instances generated. Figure 4C shows the selected coherence components from this simple algorithm for all 25 noise instances, along with a histogram that shows the number of counts generated at each frequency value (the red stem plots show the GT coherences). We observe that the FFT power spectrum does a relatively good job of recovering the correct frequency positions of the lowest frequency components, but a very poor job for the remaining components. For instance, the FFT never identifies the component at v_6_ = 12.25 THz because this term always resides below the noise threshold. It also does a poor job of recovering the frequency of the two components near 10 THz (v_4(5)_ = 10.0(10.25)THz), resulting in a distribution of frequencies across a relatively broad spectral range. Further, it also misses the v_7_ = 5.0 THz component at least 50% of the time.

Next, we examine the recovered population decay terms. Before discussing the results of MEF on the original signal, let us first look at the recovered rates in the absence of the coherence terms. This provides a means to assess the best-case scenario for MEF when it does indeed represent the correct hypothesis. Shown in Figure 4D in red are the recovered rates for a biexponential fit to the data at each noise instance along with 95% confidence intervals which may be calculated from the covariance matrix. For the slower rate at 1 THz, we see very high confidence in the recovered rate, but for the faster rate at 3.5 THz, the variance in rates is larger. If we now examine the recovered rates of the total signal which includes the coherence terms, we see that the faster rate exhibits a systematic error: . In fact, the distribution of rates recovered by MEF of the total signal does not even overlap the distribution of rates recovered for the population-only signal, . This means that the MEF/dFT approach fails to recover the true coherences and to recover the true rates, even in the presence of the low- to moderate levels of noise often encountered in ultrafast pump–probe measurements. In fact, each instance of noise leads to a different spectrum and rate which can lie outside the expected value based on the noise-variance alone. Again, this is because separating the spectral recovery problem into two steps–exponential fitting and dFT of the residual–leads to errors in both. Further, the dFT does not directly provide estimates for the line width of each feature. While each peak may be fit to a specific line shape function, this exacerbates the estimation problem by imposing an additional hypothesis (for each feature) that cannot be statistically validated. As we shall see below, even if the hypothesis is known (i.e., the line shape form, number of components, etc.), fundamental limits imposed by Fourier theory can lead to inaccurate results such as when the signal is truncated.

While the dFT does a poor job of parameter estimation with respect to both coherence and population terms, based on the results of model 2, we may expect that the LPSVD will perform significantly better. Unlike the noiseless case, however, identifying the correct rank to use in the LPSVD algorithm is now much more challenging because all the singular values become nonzero in the presence of noise. The most common way cited in the literature to identify the rank is to use the reduced chi square test,^42^ by which the correct number of components corresponds to those in which . However, this criterion introduces several problems.^43^

**Figure 5:**
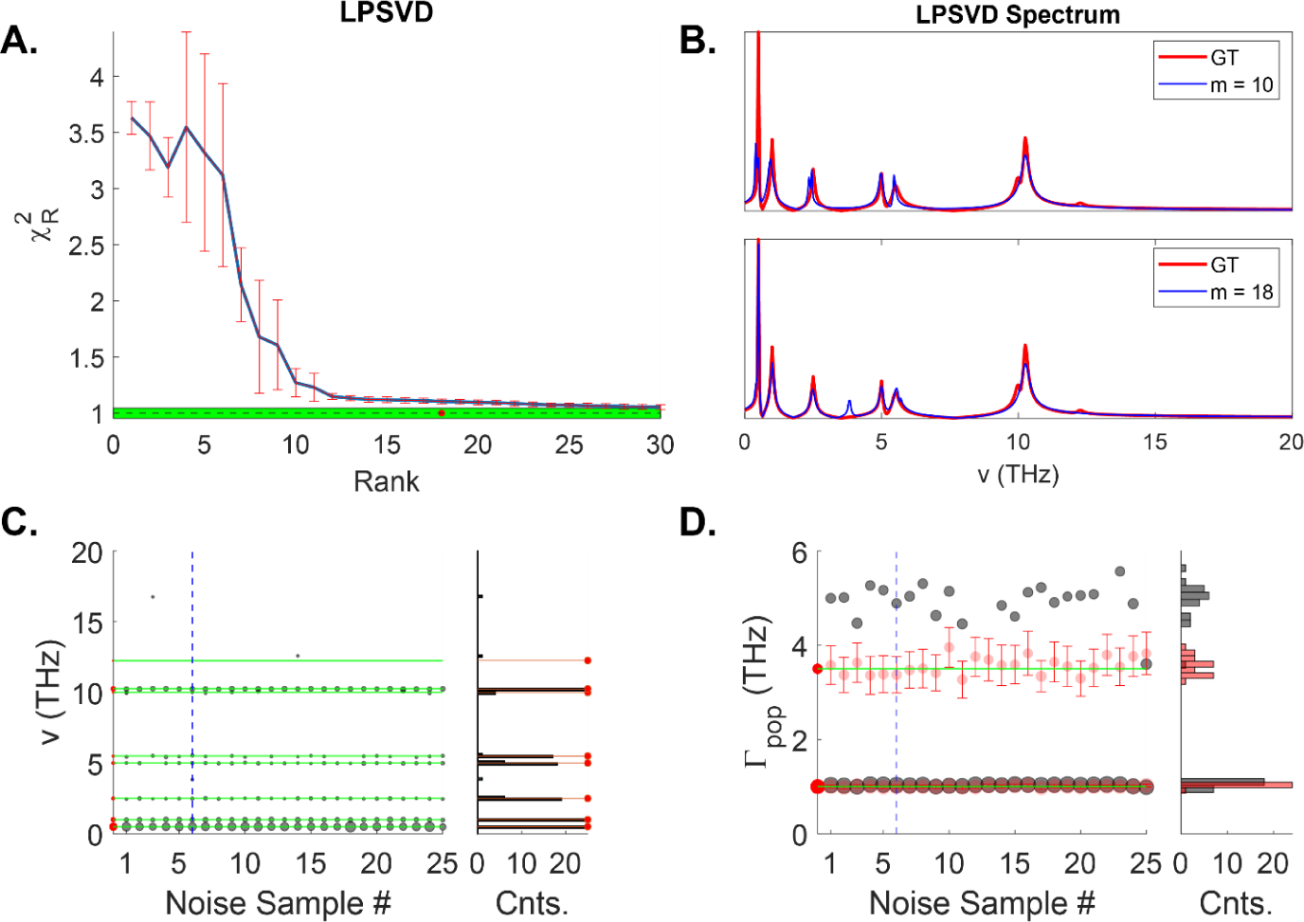
(A) LPSVD analysis on model 3 showing the reduced chi square as a function of the rank. Error bars were calculated over 25 noise instances. Green region shows the estimated chi square for optimal fitting based on the variance of the noise. Note only at the highest rank value does the calculated chi-square value fall within this range. The true rank is *M* = 18. (B) LPSVD spectra at two different rank values. (C) Left: Analysis of the detected coherences above the threshold value for 25 different noise instances. Right plot shows a histogram of the results compared to the GT frequencies and counts. (D) Analysis of the detected population rates from LPSVD. Red circles shows the results of fitting to a population-only signal (errors bars are at the 95% confidence interval). Gray circles show the decay rates of the lowest frequencies recovered from LPSVD. Histogram of both results shown on the right. The recovered rates from LPSVD are systematically different than the “true” values (red circles) for the fast component.

The first is that the definition of  requires knowledge of the number of unknowns, which is usually taken to be N – m, where N is the number of points in the signal, and m is the number of terms in the fit. But this definition only applies for linear fitting problems. However, there is a bigger issue that needs consideration, which is that  itself depends on the noise. Because each instance of noise generates a different value for the rank, one fit may be valid for one instance of noise, but not another. This issue is evident when evaluating  for each instance of noise at each rank value for the LPSVD algorithm as shown in Figure 5A. We see that indeed the mean  decreases as we increase the rank, but also that there is considerable variance at each value up to about *m* = 12, at which point the variances decreases substantially. This may occur because at this value most of the signal features are captured so that the variance of the residual lies close to the noise variance. We see a gradual decrease in the  as we further increase the rank, but only at the highest values evaluated, does  fall within the estimated variance of chi square about its mean, 2/N (note, this variance estimate only holds for the true model having the true parameter values, which cannot be the case for any case except the true rank). However, the true rank is *m* = 18, which lies somewhere in between the point at which the variance in  decreases substantially (*m* = 12) and the point at which it lies within the expected range for a given noise variance (*m* = 30). In other words, the reduced chi square test cannot correctly identify the true rank. We show two instances of the recovered spectrum (*m* = 10, *m* = 18) in Figure 5B, which correctly identify the main peaks, but miss weaker features (e.g., at 12.25 THz) as with the FFT, while generating some false peaks, including those outside the pulse bandwidth near 17 THz. As before, we may construct a histogram of the recovered coherence components for different noise samples. The recovered components strongly depend on the chosen rank, but to give a “best-case” scenario, we show the analysis only for the true rank (again, we emphasize that there is no clear way to know this value *a priori*). Indeed, we see a far better recovery than in the FFT power spectrum for most coherence components, but as with MEF, the recovered fast population decay component shows systematic error: . In fact, the MEF does a better job than the optimal LPSVD in terms of recovery of the population decay components with respect to both the mean and the variance, while optimal LPSVD does a better job in terms of coherence recovery. Yet, if we consider that the rank is unknown in most real experimental scenarios, and that the FFT requires no knowledge of it, the FFT power spectrum may be preferred for identifying the frequencies in practice. On the other hand, LPSVD does a good job of recovering the correct line widths (not shown explicitly but indicated indirectly in the amplitudes of each feature (gray circle) in Figure 5C), while the dFT does not provide any direct line width estimate.

**Figure 6:**
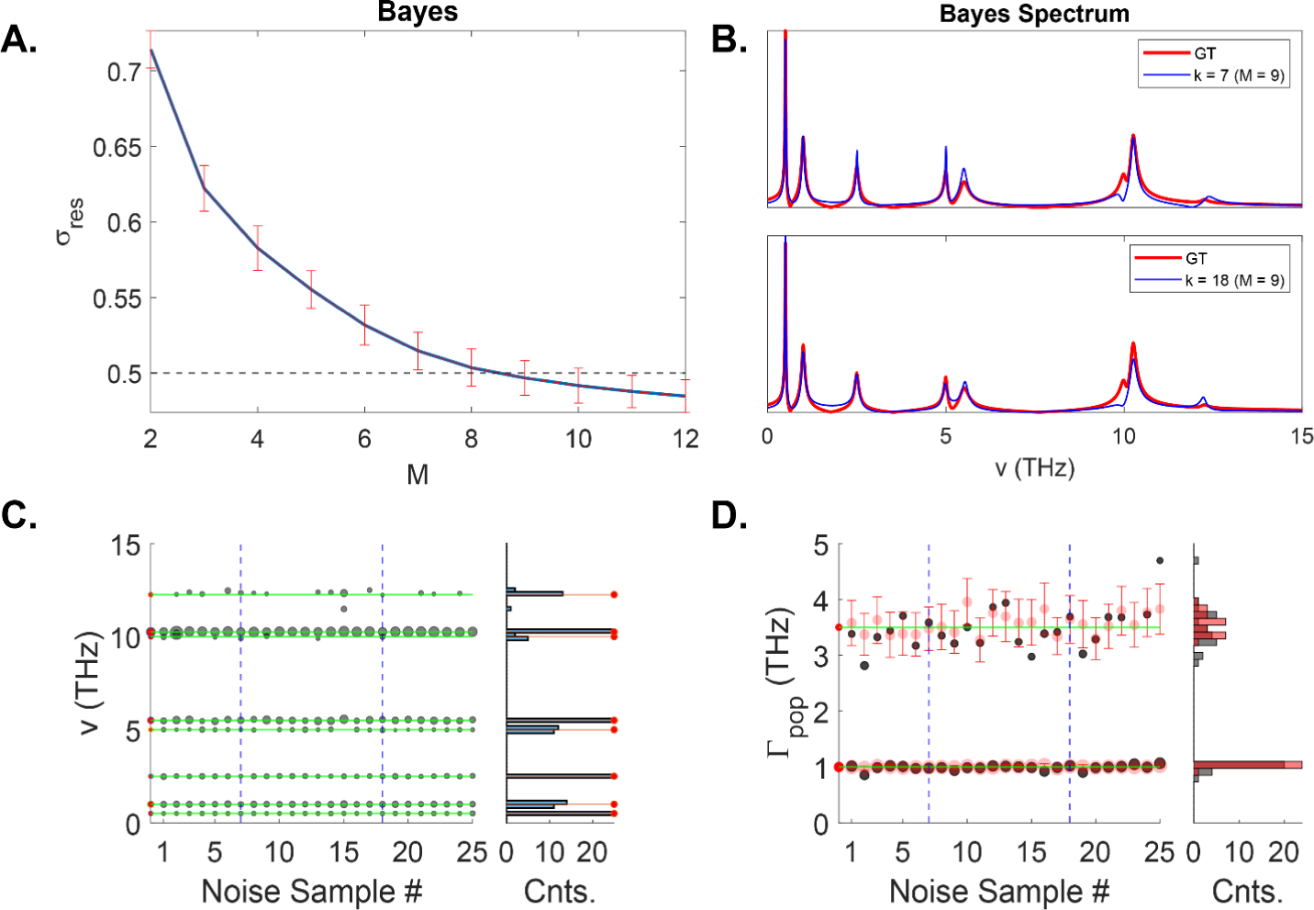
(A) Standard deviation of the Bayes reconstructed signal as a function of the number of model functions. Error bars are calculated over 25 noise instances. (B) Two representative Bayes reconstructed spectra (blue curve) and GT spectra (red curve). (C) Coherence frequencies recovered for each noise sample and corresponding histogram. (D) Population relaxation rates recovered for each noise sample (gray circles) and GT rates (red circles) and corresponding histograms.

Finally, we analyze the results of *UltraStat*. For each instance of noise, we run the optimization algorithm, evaluating the standard deviation of the residual at each iteration. For each noise instance, the algorithm terminates whenever the variance of the residual falls below the known or estimated noise variance. Typically, this occurred at *M* = 8–10 (with no instance of *M* = 11 or 12, as expected). In other words, *UltraStat* may underfit the data slightly (due to noise) but avoids overfitting it. Two instances of the recovered spectra are shown in Figure 6B, revealing excellent agreement in terms of the amplitudes, frequencies, and line widths. The most notable error appears on the shoulder of the 10 THz peak. As before, we may examine, in detail, the recovered coherence frequencies and the population decay terms for each instance of the noise. For the coherences, the recovered frequencies and amplitudes match well with the GT parameter values for all components except the weak 10 THz component. The algorithm identifies that component only about 25% of the time, but among those, the amplitudes were not well matched to the GT values. Note, the 12.25 THz component was correctly identified 60% of the time, even though it falls below the noise threshold. In addition, out of all the noise instances, in only one was an incorrect frequency component recovered (k = 15), with none occurring outside the set bounds. For the population relaxation rates, the results are even more encouraging, where we find , which is nearly identical to the nominal result, .

#### Estimating Errors

Next, we explore some features of the Bayes analysis that are not available to either the dFT or LPSVD. First, we may calculate the errors in all the parameters recovered by *UltraStat*.

**Figure 7:**
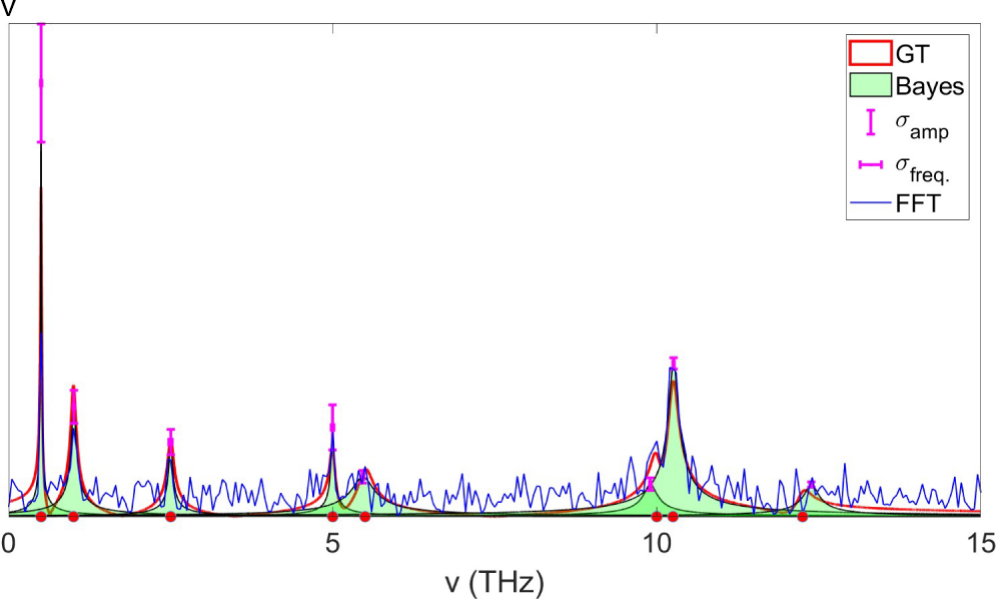
Exempler of Bayes reconstructed spectrum (green, shaded) compared to both the GT spectrum (red) and the FFT power spectrum (blue). The frequency and amplitude error bars are also displayed (plus or minus one standard deviation).

As shown in Figure 7, the errors in the frequencies and amplitudes may be estimated using the Hessian evaluated at the optimal parameters (the errors in the phase and line widths are not shown). Note, for clarity we are displaying the individual Bayes-recovered features and comparing them to the full GT spectrum which is the sum of all the coherence modes. We observe that the errors in the amplitudes are most pronounced for the lowest frequency coherence components because these are strongly coupled to errors in the recovered population decay rates. However, the errors in the frequencies for these terms are relatively small. Generally, we see larger errors for components that do not fully decay within the measurement window as expected. While all the frequencies are recovered well, the largest error occurs for the shoulder at 10 THz. This is not surprising because the presence of the main peak at 10.25 THz strongly affects the recovered frequency, line width, and phase of the nearby features. We also see a small error for the 12.25 THz peak but given its weak amplitude relative to the noise; this too is not surprising.

#### Nonuniform Sampling

**Figure 8:**
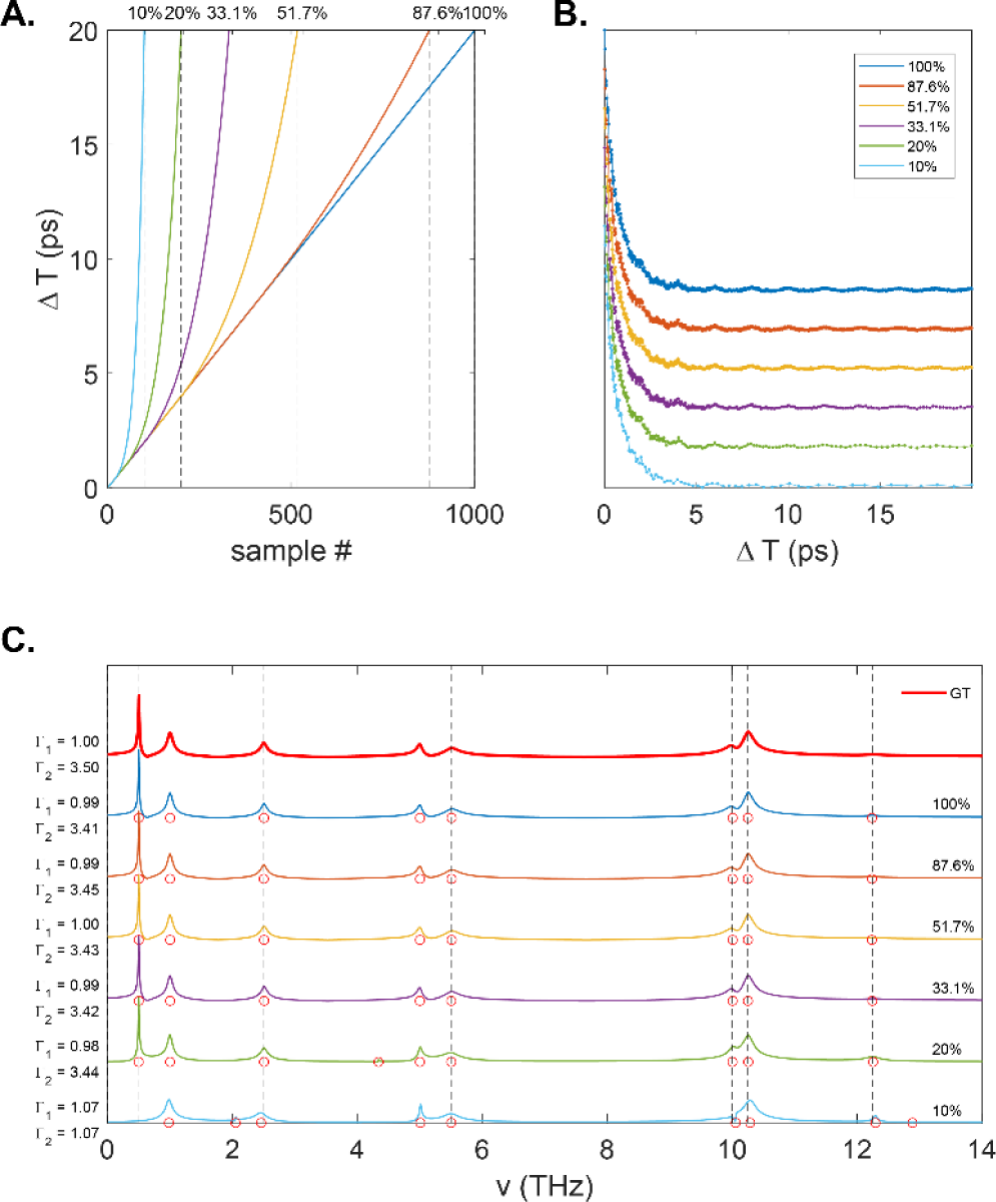
(A) Sampling schedule at different rates from 100% to 10%. (B) Time domain signals after subsampling. (C) Reconstructed Bayes spectra at different sampling rates corresponding to the schedule shown in (A). The recovered population rates are also shown on the left-hand side. Circles mark the position of the recovered frequencies. GT spectrum is shown for reference (red curve, dashed black lines, and red circles show GT frequencies).

One attractive feature of *UltraStat* (and Bayes, in general) is that it does not require uniform sampling as does both the dFT and LPSVD. The FFT is a linear transform, that requires equally spaced sampling at a rate, F_s_, sufficient to fulfill the Nyquist-Shannon theorem which states the highest frequency in the signal must be smaller than F_s_/2. The LPSVD, on the other hand, relies on the property of linear prediction, which holds true only when the sampling is uniform. Nonuniform sampling has a long history in time-domain spectroscopy, most notably in Fourier- transform nuclear magnetic resonance (FT-NMR)^44^ and is now used routinely in multidimensional NMR^45^ and in some magnetic resonance imaging (MRI) experiments.^46^ Its use in coherent optical spectroscopy is less widespread, but there have been examples in the literature using methods such as compressive sensing^47,48^ and projection reconstruction,^49^ among others. However, most of these methods ultimately utilize the Fourier transform in one way or another. In *UltraStat*, we may use knowledge about the physical response of the sample to design general sampling schedules which are highly efficient. For instance, since the signal decays in time, it is advantageous to sample more at early times than at later times, where much of the signal decays. Here, we set the sampling rate to be proportion to e^τ(j-1)^, where j is an index corresponding to the sample points and the rate, τ, controls the curvature of the sampling function. As shown in Figure 8A, adjusting the rate controls the number of sampled points, N_NUS_, and, hence, the compression ratio .

We evaluated compression ratios from 1.0 down to 0.1 of the original sampling where *N*_*t*_ = 1000. Figure 8B shows the time-domain signal for each of the sampling schedule (note, we use the same noise variance as in Model 3). We then use *UltraStat* to reconstruct the coherence spectra and recover the population decay rates for each case. In the compression-less case (η = 1), we recover the same results as before. As the compression factor increases, we observe that the correct rates are reproduced accurately up to η = 0.2, while at η = 0.1 the slower rate is accurately recovered, while the faster rate is not recovered at all. The coherence frequencies for the strongest peaks are recovered throughout the data sets, while at 10% sampling, only the lowest frequency component is missing. We note that at even lower noise levels, higher compression factors may be achieved. In a multidimensional experiment, the overall compression factor may be much higher, as sparse sampling along multiple dimensions may be utilized. In any real experiment, the limited photon budget necessitates a trade-off between the noise and the number of points sampled. Therefore, it may be more prudent to decrease the time window at the expense of spectral resolution, while increasing the compression ratio to improve the accuracy of recovery. In the context of Bayes, there is a calculated trade-off between the errors associated with each recovered parameter.

#### Super-Resolution

**Figure 9:**
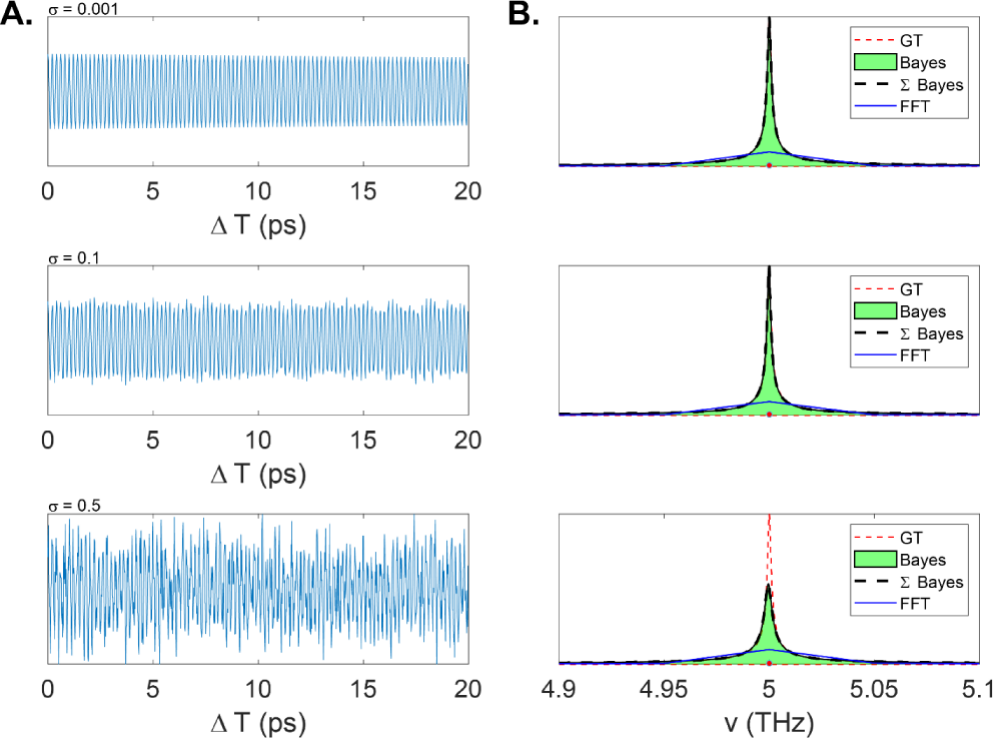
(A). Severely truncated signals at three different noise levels (0.001, 0.1, and 0.5). (B). Corresponding Bayes recovered spectra (green, shaded), GT (red dashed), and FFT (blue).

We now consider the issue of spectral resolution, which we consider from two perspectives. First, we take the case of a truncated time-domain measurement, where the Fourier limited resolution is set by the maximum time sampled, *t*_*max*_. In all the simulations explored here, *t*_*max*_ = 20 ps, giving a measurement-limited spectral resolution of 1/20 ps = 0.05 THz. Since at least three points are needed to determine the actual line width of a peak, the effective resolution is twice this value, . We now simulate a single band with a spectral line width of 0.005 THz, which is 20x below the Fourier limit. *UltraStat* is then applied to three instances of noise (σ = 0.001, σ = 0.1, and σ = 0.5, Figure 9A). As shown in Figure 9A, the signal is highly truncated, and its FFT (Figure 9B) displays a measurement-limited resolution of 0.1 THz as expected. Using *UltraStat*, the line widths in the two lowest noise cases are recovered exactly, while in the highest noise case (σ = 0.5), the recovered line width is 0.01 THz, which is twice the true value. Of course, it is also possible to perform a fit directly in the frequency domain and obtain resolution well beyond .

**Figure 10:**
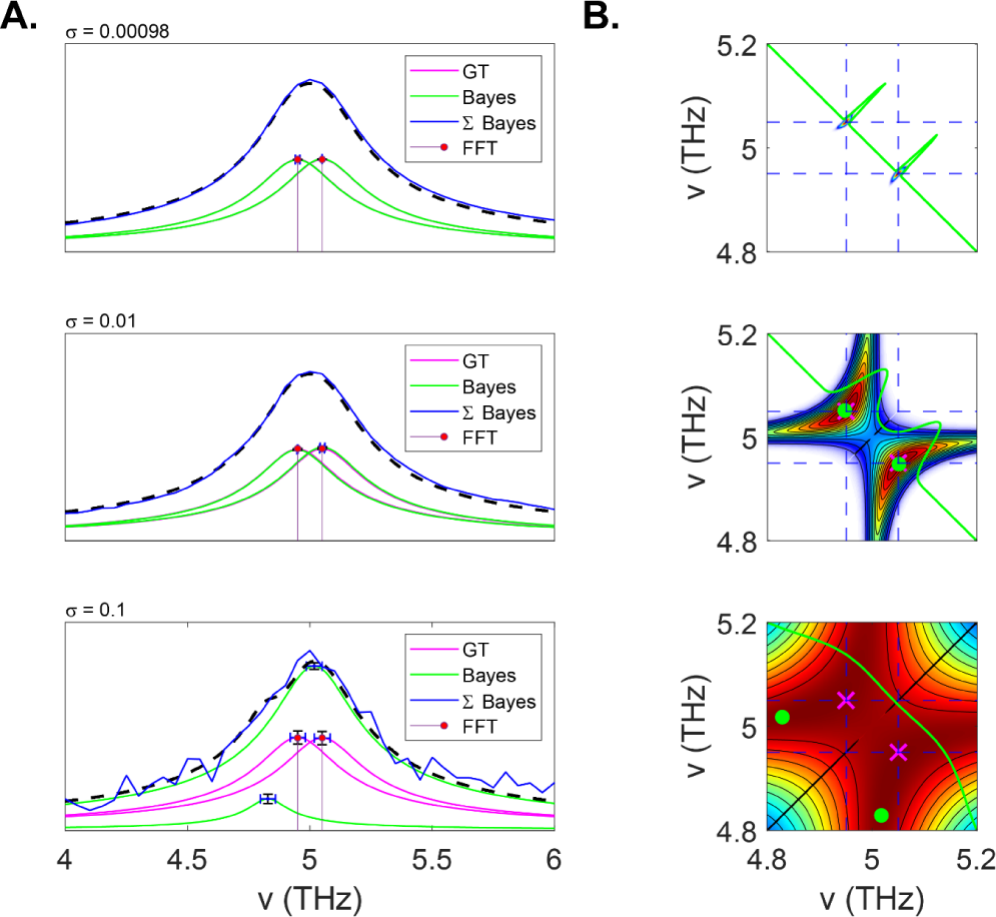
Super-resolution spectroscopy. (A) Signal in the frequency domain at different levels of noise (0.00098, 0.01, and 0.1), along with individual GT peaks (magenta) and Bayes recovered (green). Error bars for the frequency and amplitude are also shown. (B) 2D map showing the posterior probability as a function of two coherence frequencies in the 4.8–5.2 THz range. Blue-dashed lines show the GT frequency values. Green curve shows a slice of the posterior amplitude at a slide along the antidiagonal of the 2D curve. Magenta “X” shows the GT frequencies, while the green circle marks the location of the frequencies recovered by the *UltraStat* algorithm.

For well-isolated peaks, this is quite straightforward, although it requires apodization of the time-domain signal which broadens the spectrum according to the Fourier convolution theorem. At low noise values, this effect can be accounted for, and an accurate line width may be recovered. However, a more complicated case arises when separating two closely spaced features at higher noise levels. In a real experiment, the resolution is limited by noise and finite coherence times (vis a vis the line widths). Generally, at zero noise, the bands must be separated by at least their line widths to be resolvable, while noise may act to further degrade the spectral resolution. To investigate this issue further, we simulated a two-level system (TLS) where the frequency separation was set to 0.10 THz, while the line width of each transition was 1.0 THz. The amplitudes of the two modes were set equal. Figure 10A shows spectra the TLS at three different noise levels. The dFT and *UltraStat* analysis were applied to each case. At the two lowest noise values (σ = 0.001 and σ = 0.01), *UltraStat* successfully recovers the correct amplitudes, phase, dephasing rates (i.e., line widths), and frequencies. As the noise is increased further (σ = 0.1), Bayes fails to correctly identify the spectral parameters, instead yielding a primary component at the center of the two peaks (v = 5 THz), while exhibiting a small shoulder that accounts for the noisy feature of the signal on the red side of the spectrum. To better understand how resolution and noise are interconnected, we explicitly calculated the posterior probability as a function of two coherence frequencies at fixed dephasing rates and phases as shown in Figure 10B. At the lowest noise variance, the posterior is strongly peaked exactly at the two frequency positions (v = [4.95, 5.05]). The optimization algorithm easily identifies the maximum of the posterior and returns the correct parameters. At the next higher level of noise (σ = 0.01), the posterior still peaks at the correct frequency position, but now the peaks are broadened so that curvature near the extrema is flattened. At the highest noise value considered (σ = 0.1), the posterior probability further flattens in the parameter space, and locating the optimal solution becomes more difficult. *UltraStat* recovers the frequencies marked by the green circle, which is clearly not at the optimal solution (magenta “X”). This is due to the very flat topography of the posterior probability in phase space. It is interesting to note that the error bars for the recovered Bayes analysis for the highest noise case do not seem to capture the deviation of the GT signal from the recovered signal as may be expected. One reason this happens is because errors in the line widths (not shown) also couple to errors in the amplitude.

Another reason is violations of the assumptions made in calculating them. For the amplitude error calculation, the assumption was that the recovered parameters were near the optimal values, while for the parameter error calculation, the posterior probability is assumed to be quadratic with respect to the parameters. In this case, the recovered parameters are far from the optimal values so the amplitude error calculation is not accurate. Further, the quadratic assumption fails, so that higher order terms may be needed for more accurate estimates of the parameter noise. This contrasts with the errors calculated in Figure 7, were the parameters found by *UltraStat* where, in fact, very close to the true values, and the error calculations better represented the deviations from the GT values. This implies caution in assuming that the error bars encapsulate the true solution at high noise levels.

The analysis presented here addresses how noise and resolution are interconnected: noise causes flattening (loss of curvature) of the objective function near the optimal parameters, making it more difficult to locate in phase space, thereby leading to loss in resolution. While this model only considered a two-level system, the same principle holds across any number of parameter dimensions. Further, in both cases considered the model functions accurately represented the signal line shapes, while in a real experiment that true line shapes may be unknown. More general line shapes may be used (e.g., Voigt functions), at the expense of potentially more parameters.

#### Application of Priors

Bayes theorem states that prior information may act on the likelihood function to narrow the posterior. The inclusion of priors, therefore, is necessary to fully realize the potential advantages of Bayes analysis over other methods like maximum likelihood. Thus, far, we have utilized only uniform priors on the parameters to provide a more direct comparison to other methods that do not utilize priors like MEF/dFT or LPSVD. In general, utilizing priors in analysis of ultrafast experiments may be necessary when dealing with real data. First, we may consider priors on the nonlinear parameters themselves such as the frequencies, dephasing/decay rates, and phases. For instance, the coherence frequencies which arise from multiple field-matter interactions of the excitation pulse may be drawn from a known distribution with a variance determined by the pulse bandwidth. For a well-compressed pulse, the phase profile is flat across the pulse bandwidth, leading to a narrow distribution for the phase parameters. For the dephasing rate, there may be domain knowledge about the range of lifetimes of certain vibrational modes measured by frequency- or time-domain experiments. Similarly, population relaxation rates may be measured by other experiments such as those using narrowband pulses and sampling longer delay times. Depending on the specific experiment, priors may be applied to the amplitudes, such as restricting them to be only positive for signals that comprise only a single, nonlinear response pathway, or by a more complex set of bounds for experiments that integrate over multiple pathways such as ground-state bleach and excited-state absorption signals, which have opposite signs.

As a simple demonstration of the effect of employing prior information, we generated a model with a large noise variance (), whereby we compare the results of *UltraStat* with and without priors for the phase and amplitudes. Specifically, we applied a Gaussian prior to the phase parameters with  and , to simulate a flat phase response. We also added an exponential prior on the amplitudes, , to restrict the solutions to those with positive amplitudes.

**Figure 11:**
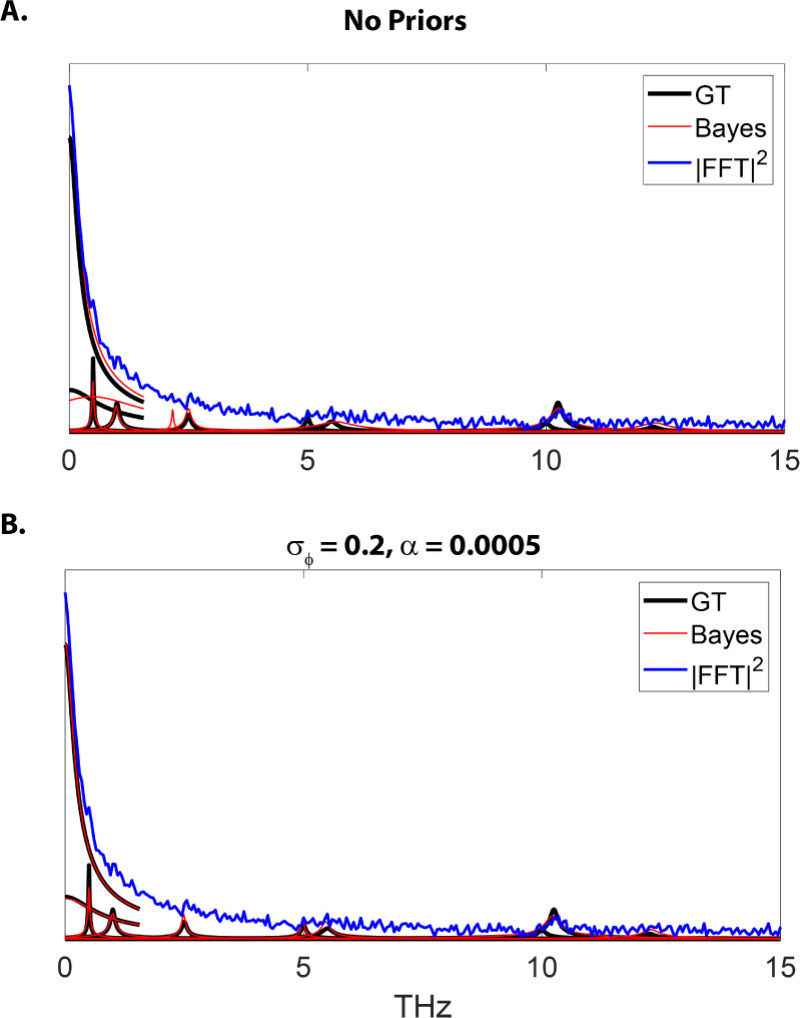
Comparison of *UltraStat* without (A) and with (B) priors. Note the exponential decay terms are truncated for easier visualization.

Therefore, the “weights” of the priors are controlled by σ_{ϕ}_ and α. These factors may be adjusted by balancing the least-squares term in the objective function against the regularization terms, which are measures of the confidence in the priors. While different procedures may be applied to systematically select these parameters, we found the results were similar for a large range of values for σ and α. However, a more systematic approach to selecting these parameters would be needed for real data (see Supporting Information for further discussion). As shown in Figure 11, the addition of these priors greatly improved parameter recovery. For instance, without priors, one of the population relaxation terms (), was mistaken for a low-frequency coherence at  with a broad line width (). The largest coherence terms were readily identified, but some of the weaker coherence terms were entirely missed (e.g., *v* = 5 THz). In contrast, when the above priors were included, all the features in the model were recovered except for the weak shoulder near . A table of the GT values and the recovered values with and without priors may be found in the Supporting Information.

## Conclusion

The first step following any ultrafast experiment is parameter estimation. Without accurate parameter estimation, the reliability of any physical interpretation of the signal origin is dubious. Unfortunately, statistical analysis is not applied in most ultrafast experiments. In the few cases where statistical analysis is performed, oftentimes it involves calculating error bars for population decay rates. Yet, as demonstrated here, such error bars have limited physical significance if the hypothesis being tested is incorrect. The effect of noise on the uncertainty in the measurement is rarely discussed or analyzed. The lack of statistical analysis, in general, may lead to erroneous interpretation of experimental results, greatly eroding confidence in ultrafast experiments despite their growing prominence in the physical sciences.

Here, we have outlined a general methodology based on probability theory which we call *UltraStat* that takes advantage of knowledge about the signals being generated. *UltraStat* provides a robust method to fit complex time-resolved data over a very large number of dimensions, capturing both the coherence and population signals simultaneously. By treating all the signal components on the same footing, *UltraStat* overcomes the limitations inherent in mixed MEF/dFT analysis. Notably, it provides error bars to all parameters and amplitudes, which is critical for assessing the statistical significance of these parameters across different experiments. For instance, *UltraStat* could answer the question of whether making a structural modification to a molecule under study changes the frequency, amplitude, or line width of a particular mode with statistical significance. As demonstrated here, one of the most troubling aspects of MEF/dFT and other methods such as LPSVD is that they retrieve the incorrect population decay rates. As the recovered rates are extremely sensitive to the frequency and amplitude of low-frequency coherences, modification of the sample or experimental conditions (e.g., the pump or probe wavelength) that leads to changes in these components will lead to fits with very different decay rates even if the true rates remain largely unchanged. *UltraStat*, on the other hand, fits both coherence and population terms simultaneously, so that changes in both are accurately captured. A key feature of Bayes analysis is the use of prior information. We have demonstrated a simple example on how priors may be applied, but a deeper analysis is needed for analyzing real experiments with more complex constraints. In addition to the parameters and amplitudes, priors may also be applied to knowledge of the noise, such as its distribution (e.g., Gaussian noise), whereby the most probably noise variance may be estimated by Bayesian analysis or where a known variance can be used to select a particular hypothesis as was done here. If the noise exhibits a more complex distribution which may be independently measured, this too could be incorporated as prior information. A powerful feature of Bayes theory is that the posterior from one data set may be used as a prior for another data set. This strategy could be used, then, to understand a series of experiments where parameters are only changed slightly over time but may still be informed by prior observation, thereby combining prior knowledge and new data in a consistent and principled way. Future work on this topic is forthcoming.

Another key advantage of *UltraStat* is that it is not bound by the limitations of Fourier theory. While the continuous FT can accurately recover the spectrum of a continuous time-domain signal, the dFT deviates from the true spectrum in any real experimental scenario, becoming more distorted in the presence of large population signals and noise, or when the signal is truncated. While apodization can eliminate truncation artifacts, it is not possible to find a matched filter function to all the signal components simultaneously. This results in varying magnitude of distortions for different modes. As low-frequency coherences may have long dephasing times, while high-frequency coherences may have short dephasing times, signals generated with broad bandwidth pulses are difficult to analyze with Fourier methods. Here, we show that *UltraStat* can recover accurate line shapes even when the signal is highly truncated, by more than an order-of-magnitude beyond the limits of Fourier theory. Further, *UltraStat* can distinguish two closely spaced features beyond the time-bandwidth product limits, by as much as an order-of-magnitude. Importantly, these limits are set not by the time-bandwidth product, but rather by the level of noise in the measurement. Therefore, noise plays a critical role in spectral resolution. It is important to point out that knowledge of the line shape can play a large role in the ability to achieve resolution advantages over Fourier methods. Incorrect assumptions about the line shape may severely degrade performance. This issue is a topic of ongoing research and will be explored in future work.

The role of noise in spectral resolution puts experimental efforts to surpass the Fourier limit in a different perspective. For instance, several proposals and experiments using the quantum nature of light have demonstrated spectral resolution surpassing the traditional Fourier limits,^37,50,51^ This is possible because the energies of the down-converted photons are correlated in a quantum mechanical sense (e.g., they violate Bell’s inequality). While theoretically, the use of entangled photons can surpass the Fourier limited resolution set by the time-bandwidth product, the experimentally achievable resolution will strongly depend on the noise in the measurements. In most implementations, such quantum measurements are inherently noisier because they involve using only a single photon at a time to maintain the quantum correlations. This necessitates many more individual measurements, which may be challenging due to photobleaching or photodamage, in general. If indeed these quantum measurements are accompanied by higher levels of noise, the uncertainty in the retrieved parameters may exceed those of their classical counterparts, negating their theoretical advantage. A similar argument holds for single-molecule or single-particle measurements,^14,15,52,53^ which eliminate the effects of inhomogeneous broadening, but may be associated with higher levels of noise. If the added noise leads to greater parameter uncertainty, not only are the advantages of these measurements reduced, but the uncertainties in the parameters may exceed the corresponding ensemble-averaged estimates.

Another advantage of *UltraStat* is the utilization of nonuniform sampling. In many cases there is a limited photon budget which imposes severe restrictions on the number of points sampled in the time-domain, the ability to perform signal averaging, etc. Subsampling could be a powerful method to circumvent the photon budget limitation, but accurate spectral recovery is challenging. For instance, using compressive sensing (CS), the sparsity of the spectrum may be exploited to reduce the sampling requirements. However, CS would be challenging to perform on signals that contain both coherences and populations as a sparse domain may not be available. Further, there is no objective means by which to select the compression ratio, or to determine if the results are accurate on an unknown sample. As CS typically uses a pseudorandom sampling schedule,^54^ each implementation may lead to different results, making the approach far less general. With *UltraStat*, the recovery is robust to the sampling schedule, while statistical tests with respect to the noise variance are possible. Here, we have shown spectral recovery of noisy signals down to less than 10% of the Nyquist-limited sampling rate. As with the case of spectral resolution, the results are highly dependent on the noise variance, with higher or lower compression ratios possible. We note that in multidimensional experiments, the subsampling approach may lead to even high compression ratios, as the results scale with the number of dimensions. In a 2D experiment, for instance, the sampling may be reduced by as much as 99% at lower noise values. The sampling schedule may be further optimized using prior knowledge of the frequencies or decay rates, if available. For instance, in experiments where only a few features are analyzed, the schedule could be selected to recover those features preferentially, greatly reducing the sampling requirements even beyond what was demonstrated here. While not explicitly analyzed here, subsampling may be necessary for large ultrafast microscopy experiments,^33,55^ where the demands on photon budget are even greater.

While the model functions used here were Lorentzian to allow comparison to LPSVD, other functions could be used as well (e.g., Voigt, Gaussians, etc.) that better capture the expected line shapes. Adapting *UltraStat* to other line shapes only requires evaluating the derivatives either numerically or, preferably, analytically. It is important to point out that the model functions do not necessarily have to represent the true line shapes of the modes found in the system; rather, they are simply basis functions that best represent the signal mathematically. If, for example, a line shape exhibits non-Lorentzian behavior due to a vibronic progression, then more than a single basis function may be needed. It remains up to the practitioner to determine the physical basis for any representation; that is, to determine the correct that may best describe the line shape function. If the user determines that each mode is inhomogeneously broadened, for instance, then a Gaussian convolution may be imposed on the model functions. More generally, *UltraStat* allows any knowledge about the system to be systematically employed in improving the parameter estimation problem–a key feature not available to other methods. Future work will focus on selecting optimal model functions including the use of more sophisticated performance metrics such as Bayesian information criteria (BIS), the use of more complex noise functions, and the incorporation of Monte Carlo sampling methods for with large parameter space.

In summary, *UltraStat* is a powerful method to analyze time-domain spectroscopy experiments. By using prior information about the signals, parameter estimation can be greatly improved, beyond methods that make no such assumptions (e.g., dFT). Currently, the results of different ultrafast experiments cannot be directly compared in many cases owing to differences in the experimental setup, light sources, noise levels, detectors, etc. *UltraStat* would enable comparisons among different measurements quantitatively while providing uncertainties in the recovered parameters. In a future work, we will investigate other model functions and additional complexity that may be encountered in real experiments such as non-Gaussian noise from laser intensity and phase fluctuations, sample constraints, etc. A Bayesian approach could prove important for revealing otherwise hidden interactions and physical processes using a systematic and probabilistic approach. Further, the use of *UltraStat* to analyze multidimensional spectroscopy and imaging experiments may greatly improve sensitivity and resolution over the current state-of-the-art.

## Data Availability

The basic *UltraStat* code in the Matlab programming environment is available at https://github.com/elharel/UltraStat.

## Supporting Information Available

Theoretical description of estimating errors, details of the optimization algorithm, additional details on the use of priors, and derivations of some equations (PDF)
