# Supplementary material for: UltraStat: Ultrafast Spectroscopy beyond the Fourier Limit Using Bayesian Inference
Source: J Phys Chem A. 2024 Oct 16;128(42):9323–36. doi: 10.1021/acs.jpca.4c04385 (PMC11514019; doi:10.1021/acs.jpca.4c04385)
Supplement: Supplementary file 1 — jp4c04385_si_001.pdf [file jp4c04385_si_001.pdf]

Supplementary Information

UltraStat - Ultrafast Spectroscopy Beyond the Fourier Limit using Bayesian Inference

Elad Harel

Department of Chemistry, Michigan State University, 578 South Shaw Lane, East Lansing MI 48864

Corresponding Author

elharel@msu.edu

Estimating Errors. Once the optimal parameters are found, the next step is to estimate the errors of the recovered parameters and amplitudes. Starting with the latter, the probability distribution of the amplitudes given the prior information is,

$$P(B \mid I) \propto e^{-Q/2\sigma^2} \tag{1}$$

where we have assumed that the parameters are very near to the true values, $\mathbf{\Phi}_{\mathrm{opt}}$. If, in fact, we are far from the true parameters, the amplitude errors may be more difficult to evaluate without knowledge of $\mathrm{P}(\mathbf{\Phi} \mid \mathrm{I})$. To determine the error in the amplitudes, we need to calculate,

$$\delta_{\mathrm{k}}^2 = \langle \mathrm{B}_{\mathrm{k}}^2 \rangle - \langle \mathrm{B}_{\mathrm{k}} \rangle^2 \tag{2}$$

The amplitudes may then be reported to within one standard deviation as $\mathrm{B}_{\mathrm{k}} = \mathrm{B}_{\mathrm{k,opt}} \pm \delta_{\mathrm{k}}$. The

averages could be computed directly by evaluating the appropriate integrals, but the form shown in equation (19) is not convenient because the amplitude terms are coupled. We may separate the coupled integrals into a product of independent Gaussian integrals by applying the appropriate change of variable (see Appendix II). The result is,

$$\delta_{\mathrm{k}}^2 = \sigma^2 \sum_{\mathrm{l}} \frac{\mathbf{e}_{\mathrm{lk}}^2}{\lambda_{\mathrm{l}}} \qquad (3)$$

where the vectors, $\mathbf{e}_{\mathrm{j}}$, are eigenvectors and λ_{j} are the corresponding eigenvalues of the matrix, $\hat{g}$, whose matrix elements are the inner product of the model functions with respect to time,

$$g_{jk} = \mathrm{M_j} \cdot \mathrm{M_k} \qquad (4)$$

For estimating the parameter errors, we cannot use the same approach because of the nonlinear form of the model functions. Instead, we can approximate the errors by employing a Taylor expansion of $\tilde{\mathrm{Q}}$ around the optimal parameters,

$$\tilde{\mathrm{Q}} \approx \frac{1}{2} \sum_{\mathrm{j,k}} \frac{\mathrm{d}^2\tilde{\mathrm{Q}}}{\mathrm{d}\boldsymbol{\Phi}_{\mathrm{j}}\mathrm{d}\boldsymbol{\Phi}_{\mathrm{k}}} \Delta\boldsymbol{\Phi}_{\mathrm{j}}\Delta\boldsymbol{\Phi}_{\mathrm{k}} \qquad (5)$$

where $\Delta\boldsymbol{\Phi}_{\mathrm{j}} \equiv \boldsymbol{\Phi}_{\mathrm{j,opt}} - \boldsymbol{\Phi}_{\mathrm{j}}$ (hence, the linear term in the expansion is zero). Using the same change-of-variable approach as with the amplitudes (see Appendix II), we may evaluate,

$$\gamma_{\mathrm{k}}^2 = \langle \boldsymbol{\Phi}_{\mathrm{k}}^2 \rangle - \langle \boldsymbol{\Phi}_{\mathrm{k}} \rangle^2 \qquad (6)$$

to yield

$$\gamma_k^2 = \sigma^2 \sum_l \frac{u_{lk}^2}{v_l} \quad (7)$$

where the vectors, $\mathbf{u}_j$, are eigenvectors and v_j are the corresponding eigenvalues of the matrix, $\hat{H}$, whose matrix elements are the second derivatives (i.e. the Hessian),

$$H_{jk} = \frac{1}{2} \frac{d^2\tilde{Q}}{d\boldsymbol{\Phi}_j d\boldsymbol{\Phi}_k}\bigg|_{\Phi_{opt}} \quad (8)$$

Optimization. Up to this point, the combination of VP and Bayes theory frames the parameter estimation problem in terms of maximizing the posterior probability, $P(\boldsymbol{\Phi},B \mid D,I)$, which may be directly calculated for each value of $\boldsymbol{\Phi}$. The high cost to this approach, however, compared to linear transforms such as the FFT is that this amounts to a very large optimization problem over potentially tens to hundreds of parameters. While marginalization of the amplitudes greatly reduces the dimensionality of the search, it remains nontrivial to perform a global optimization over such a large phase space. Despite this limitation, advances in optimization methods with respect to speed and accuracy have dramatically narrowed the gap in speed compared to simple transforms, without the disadvantages of poor parameter estimation.

The optimization algorithm starts by defining the objective function as the negative of logarithm of the posterior,

$$\chi^2(\boldsymbol{\Phi}) = \frac{\tilde{Q}(\boldsymbol{\Phi})}{2\sigma^2} + f_B(\boldsymbol{\Phi}) + g(\boldsymbol{\Phi}) \quad (9)$$

where $f_B(\boldsymbol{\Phi}) \equiv -\log P(B \mid \boldsymbol{\Phi},I)$ and $g(\boldsymbol{\Phi}) \equiv -\log P(\boldsymbol{\Phi} \mid I)$. The last two terms on the right-hand side of equation (27) are equivalent to regularization terms in the language of ill-

conditioned inverse problems which take advantage of some prior knowledge to stabilize the solution. Common regularization terms for the amplitudes are sparsity[1], Tikhonov[2], and total variational (TV)[3]. For the parameters, additional information such as the phase profile of the pulses or the bounds set by the pulse bandwidth may be used to define a regularization function.

The optimization is broken up into two steps: 1) a global search procedure to locate regions close to the global minimum, and 2) a local search procedure based on gradient descent to converge to the optimal solution. The former can be accomplished by one of many different types of established algorithms such as simulated annealing, genetic algorithms, etc. We will not survey these here. For all the fittings, the global search was carried out using a simulated annealing algorithm (Mathworks Matlab 2023a, Global Optimization Toolbox) and the local search was carried out using constrained nonlinear optimization with a trust-region reflective algorithm (Mathworks Matlab 2023a, Optimization Toolbox). The local optimization routine used analytical forms for the Jacobian (first derivative) and Hessian (second derivative) of χ^2 to speed up the search. Since both $\tilde{\mathrm{Q}}$ and the priors, $\mathrm{P}(\mathrm{B}\,|\,\boldsymbol{\Phi},\mathrm{I})$ and $\mathrm{P}(\boldsymbol{\Phi}\,|\,\mathrm{I})$ depend on the parameters and amplitudes, we must evaluate the derivatives of each directly. In the expression for $\tilde{\mathrm{Q}}$ given in (5), only T explicitly depends on $\boldsymbol{\Phi}$. The gradient of T is,

$$\nabla\hat{\mathrm{T}} = 2\nabla\hat{\mathrm{M}}\hat{\mathrm{G}}^{-1}\widehat{\mathrm{M}^{\dagger}} - \hat{\mathrm{M}}\nabla\hat{\mathrm{G}}\hat{\mathrm{G}}^{-1}\widehat{\mathrm{M}^{\dagger}} \tag{10}$$

where $\hat{\mathrm{G}} \equiv \widehat{\mathrm{M}^{\dagger}}\hat{\mathrm{M}}$. The Hessian of T is

$$\begin{aligned}\nabla^2\mathrm{T} = 2(&\nabla^2\mathrm{M}\mathrm{G}^{-1}\mathrm{M}^{\dagger} + \nabla\mathrm{M}\nabla\mathrm{G}^{-1}\mathrm{M}^{\dagger} + \nabla\mathrm{M}\mathrm{G}^{-1}\nabla\mathrm{M}^{\dagger}) \\ &+ (\nabla\mathrm{M}\nabla\mathrm{G}^{-1}\mathrm{M}^{\dagger} + \mathrm{M}\nabla^2\mathrm{G}^{-1}\mathrm{M}^{\dagger} + \mathrm{M}\nabla\mathrm{G}^{-1}\nabla\mathrm{M}^{\dagger})\end{aligned} \tag{11}$$

Therefore, for the Jacobian and Hessian of the objective function, we get,

$$\nabla\chi^2(\mathbf{\Phi}) = -\frac{S(q,t)\cdot\nabla\hat{T}S(q,t)}{2\sigma^2} + \nabla f_B(\mathbf{\Phi}) + \nabla g(\mathbf{\Phi}) \tag{12}$$

and

$$\nabla^2\chi^2(\mathbf{\Phi}) = -\frac{S(q,t)\cdot\nabla^2\hat{H}S(q,t)}{2\sigma^2} + \nabla^2 f_B(\mathbf{\Phi}) + \nabla^2 g(\mathbf{\Phi}) \tag{13}$$

After performing a local search, the signal is reconstructed according to equation 6 (main manuscript) and the residual is calculated, $S_{res}(t) = S(t) - S_{rec}(t)$. Depending on whether the sampling is uniform or non-uniform, the dFT of the residual is calculated either using the FFT algorithm or the non-uniform FFT algorithm[4]. The maximum in the residual spectrum is selected as a starting point for another global search, in which the objective function is now defined with respect to the residual instead of the original signal. The hypothesis now being tested is one in which a single model function best fits the residual signal. The result of the search is updated nonlinear parameters, which are used in the next iteration of the algorithm. In this way, after each iteration the largest component of the signal is eliminated and used for generating the initial point in the next instance. It is important to note that the main search algorithm always applies to the original signal to fit all the parameters simultaneously, while the procedure above is only used for identifying a good starting point for each iteration. The variance of the residual is then compared to the known or estimated noise variance. When the residual variance falls below the noise variance, the algorithm terminates. The signal is then reconstructed using the optimal

parameters and the errors are calculated for both the parameters and amplitudes from the Hessian.

Speed. While Bayes inference has been long appreciated as a powerful method for parameter estimation, including in spectral recovery problems, the major drawback of the method has been speed. The FFT is remarkably fast, requiring only a few microseconds per data point on a single core processor. LPSVD is far slower because it requires calculation of the SVD. For moderate size data sets ($\mathrm{N} < 1000$), the calculation is rather fast (see below), but as the data size increases, the SVD imposes a severe limitation. While typically not an issue for one dimensional spectroscopic data, the LPSVD is unlikely to be suitable for large multi-dimensional data sets such as those encountered in spectrally resolved pump-probe, multi-dimensional spectroscopy, and ultrafast imaging. *UltraStat* is inherently slower than the FFT, but for large data sets, typically faster than LPSVD. While *UltraStat* does not require storing large matrices as required by the SVD, it is an iterative method which imposes certain speed limitations. For the data sets encountered here, *UltraStat* required <10 seconds to execute on a multi-core laptop. Certainly, faster implementations could be realized, where computing times were reduced to the sub-second regime. However, speed is not a serious practical limitation in most scenarios since spectral recovery is not the rate limiting step in most ultrafast experiment that includes data acquisition, storage and readout, signal processing, spectral calibration, or interpolation, etc.

To more quantitatively compare CPU times, we performed *UltraStat* on a simulated signal with 10 modes (30 parameters in total), varying the indirect dimension of the time-domain signal, which is represented by the index ‘q’ (see theory section in main manuscript). Specifically, we

made ‘R’ copies of the time-domain signal, S(t) to generate an N x R matrix to be fed into the *UltraStat*. The number of copies varied from 1 to 1000, while N = 1000 was held constant. Therefore, the number of data points varied from 10^3 to 10^6. The CPU times are shown below.

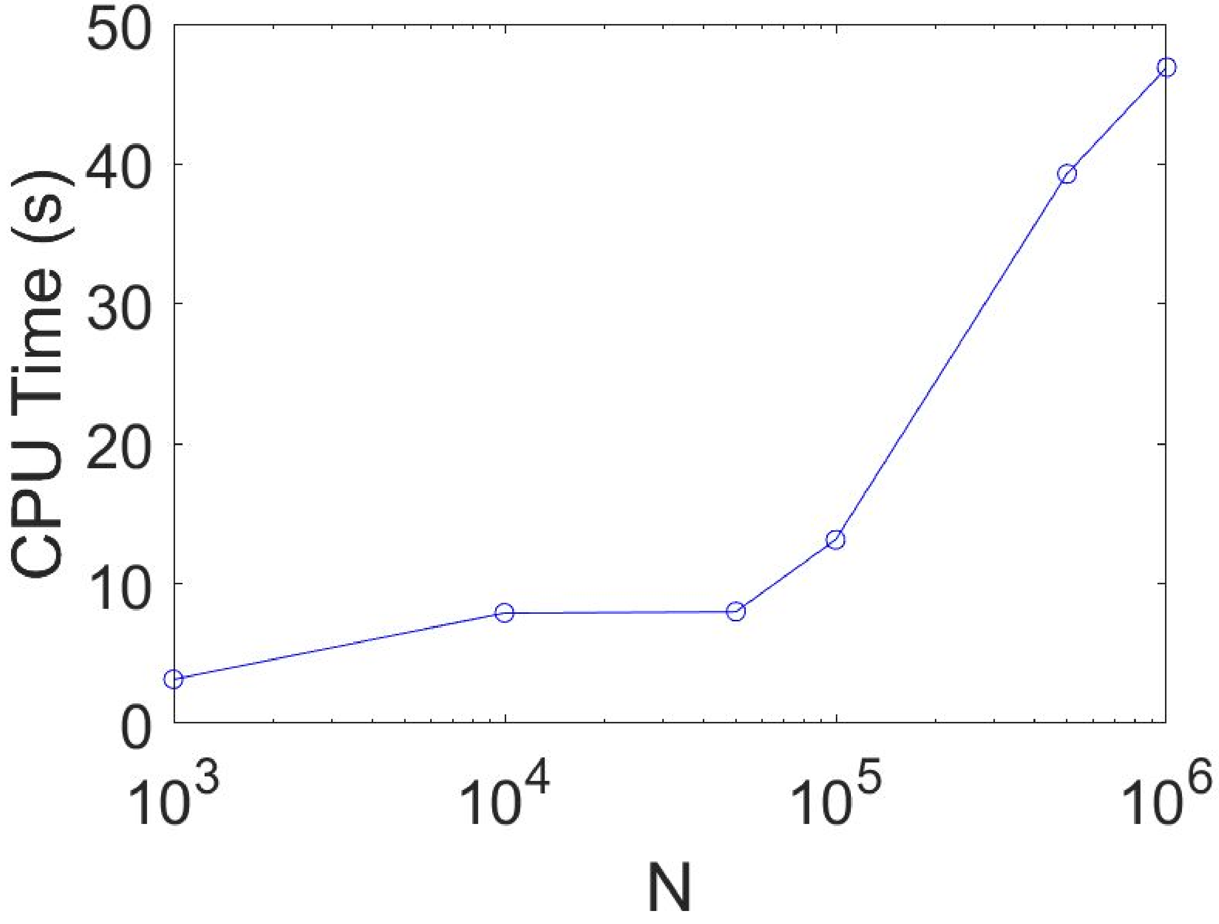

Figure S1. CPU time of *UltraStat* as a function of the total number of points for M = 10 modes (30 nonlinear parameters).

The CPU time dependence on the nonlinear parameters, M, is more difficult to quantify because it depends in a nonlinear way on details of the optimization space and the parameters chosen in the local and global search optimization algorithms. For most cases analyzed, we find CPU times for Nt = 1000, to be <10 seconds up to M = 20 (60 parameters). For large parameter space

optimization, it may be necessary to use more advanced optimization algorithms which incorporate Monte Carlo sampling methods.

Use of Priors. Details of the simulation presented in Figure 11 are provided in the tables below:

Table S1. Ground-truth (GT) values. $\sigma^2_{\{noise\}} = 1.0$

v	0	0	0.5	1.0	2.5	10.0	10.25	12.25	5.0	5.5
Γ	1	3.5	0.05	0.25	0.3	0.5	0.5	0.7	0.25	0.7
Φ	0	0	0	0	0	0	0	0	0	0
B	40	20	0.5	1	0.7	0.6	2.0	0.5	0.5	1.0

Table S2. Recovered values using UltraStat (no priors)

v	0	0.48	0.5	0.98	2.5	2.16	10.26	12.25	[redacted]	5.59
Γ	1.03	6.0	0.1	0.27	0.35	0.11	0.5	0.7	[redacted]	0.7
Φ	3.55	2.06	0	0.31	2.99	4.88	0	0	[redacted]	0
B	-47.0	-28.8	0.69	0.96	-1.11	0.31	3.03	2.05	[redacted]	3.07

Table S3. Recovered values using UltraStat (with priors, $\sigma = 0.2$ *and* $\alpha = 0.005$

v	0	0.05	0.5	1.0	2.49	[redacted]	10.25	12.28	4.99	5.46
Γ	0.99	3.69	0.09	0.35	0.22	[redacted]	0.49	1.02	0.18	0.48
Φ	0	0	0	0	0	[redacted]	0	0	0	0
B	40.24	19.55	0.68	1.20	0.75	[redacted]	1.81	1.32	0.48	1.16

It is important to note that if we select different parameters for the priors, we may get different

results. For instance, if we increase the amplitude prior by x10, the phase terms for the population relaxation terms deviate from 0, which causes errors in the amplitudes. Therefore, the phase prior should be dominant if we have high confidence that the phase is flat. This constraint gives better results for the amplitudes, as well because they are partially coupled to the phase terms according to the form of the model functions (for instance, a phase change of pi is equivalent to a sign change in the amplitude). If instead, we choose a x10 higher standard deviation for the phase terms, we get very similar results except the component at 5 THz is now absent.

In general, selecting the weights of priors in Bayesian analysis involves choosing how much influence the prior knowledge or beliefs have on the posterior distribution relative to the observed data. This requires careful consideration of the level of certainty in the prior information. Strong priors with narrow distributions are used when there is high confidence in the prior knowledge, effectively giving more weight to the prior and less to the data. Conversely, weak or uninformative priors with broad distributions are chosen when little is known about the parameters in advance, allowing the data to have greater influence on the posterior. There are many methods to select the strength of the priors including using data-driven approaches, cross-validation (if training data is available), the use of reference priors (e.g. Jeffreys prior), sensitivity analysis, among many others.

Appendix

I. Derivation of Equation 5:

We start by writing Q as follows,

$$Q = \sum_{i=1}^{N_t}\sum_{j=1}^{N_r}\left(S_{ij} - \sum_{k=1}^{M} M_{i,k}B_{k,j}\right)^2$$

The amplitudes, $B_{k,j}$, that minimize Q must satisfy,

$$\frac{\partial Q}{\partial B_{k,j}} = 0,\ \ k = 1,2,\ldots,M\ \ j = 1,2,\ldots,N_r$$

When differentiating, the only terms that survive are those that contain the term $B_{k,j}$

$$0 = \sum_{i=1}^{N_t}\left(S_{i,j} - \sum_{k'=1}^{M} M_{i,k'}B_{k',j}\right)M_{i,k}$$

Rearranging, we get,

$$\sum_{i=1}^{N_t} S_{i,j}M_{i,k} = \sum_{i=1}^{N_t}\sum_{k'=1}^{M} M_{i,k'}B_{k',j}M_{i,k}$$

Since this is true for all $k = 1,2,\ldots,M$ and $j = 1,2,\ldots,N_r$, we can sum over all these terms and multiply each by $B_{k,j}$

$$\sum_{k=1}^{M}\sum_{j=1}^{N_r}\sum_{i=1}^{N_t} S_{i,j}M_{i,k}B_{k,j} = \sum_{k=1}^{M}\sum_{j=1}^{N_r}\sum_{i=1}^{N_t}\sum_{k'=1}^{M} M_{i,k'}B_{k',j}M_{i,k}B_{k,j}$$

Substituting back into the expression for Q

$$Q = \sum_{i=1}^{N_t}\sum_{j=1}^{N_r}\left(S_{ij}^2 - \sum_{k=1}^{M} S_{i,j}M_{i,k}B_{k,j}\right) = |S(r,t)|^2 - S(r,t)\cdot\left[\hat{M}B(r)\right]$$

We can recover the amplitudes using the pseudo-inverse:

$$B(r) = \widehat{M^+}(\boldsymbol{\Phi},t)S(r,t)$$

where $M^+ = (M^\dagger M)^{-1}M^\dagger$. Substituting this expression into X gives the final expression:

$$Q = |S(r,t)|^2 - S(r,t)\cdot\left[\hat{T}S(r,t)\right]$$

where

$$\hat{T} \equiv M(M^{\dagger}M)^{-1}M^{\dagger}$$

II. Derivation of equations (3) and (7):

We start by calculating the error for the amplitudes. Assuming we have found the optimal parameters, $\mathbf{\Phi}_{\mathrm{opt}}$, then the probability of the amplitudes given the prior information is

$$P(B\,|\,I) \propto e^{-Q/2\sigma^2}$$

where $Q = |S - MB|^2$. Therefore,

$$P(B\,|\,I) \propto e^{-(|MB|^2 - 2S\cdot MB)/2\sigma^2} \equiv e^{-Q''/2\sigma^2}$$

This form is not convenient for integration because the B terms are coupled. We can rearrange the equation so that,

$$Q'' = -2\sum_{j=1}^{M} B_j S\cdot M_j + \sum_{j,k=1}^{M} B_j B_k g_{jk}$$

where $\hat{g}$ is defined in equation 22. Since $\hat{g}$ is a symmetric matrix, its eigenvectors are orthogonal. It is straightforward to show that,

$$\sum_{k=1}^{M}\sum_{l=1}^{M} e_{jl} g_{kl} e_{ik} = \lambda_i \delta_{ij}$$

where e_{jl} is the lth element of the jth eigenvector of $\hat{g}$. If we now define new variables, A, such that

$$B_k = \sum_{l=1}^{M} \frac{A_l e_{lk}}{\sqrt{\lambda_l}}$$

Substituting this into Q″, we find

$$Q'' = -2\sum_{j=1}^{M} A_l h_l + \sum_{l=1}^{M} A_l^2$$

where we have defined

$$h_l \equiv \sum_{j=1}^{M} \frac{e_{lj}}{\sqrt{\lambda_l}} \hat{g} M_j$$

We can now calculate the average and variance of the new variables A_j:

$$\langle A_j \rangle = \frac{\int_{-\infty}^{\infty} A_j e^{-\left(A_j^2 - 2h_j A_j\right)/2\sigma^2}\, dA_j}{\int_{-\infty}^{\infty} e^{-\left(A_j^2 - 2h_j A_j\right)/2\sigma^2}\, dA_j} = h_j$$

and

$$\left\langle A_j^2 \right\rangle = \frac{\int_{-\infty}^{\infty} A_j^2 e^{-\left(A_j^2 - 2h_j A_j\right)/2\sigma^2}\, dA_j}{\int_{-\infty}^{\infty} e^{-\left(A_j^2 - 2h_j A_j\right)/2\sigma^2}\, dA_j} = \sigma^2 + h_j^2$$

Using the relationship between A_j and B_k, we find that

$$\delta_k^2 \equiv \langle B_k^2 \rangle - \langle B_k \rangle^2 = \sigma^2 \sum_{l=1}^{M} \frac{e_{lk}^2}{\lambda_l}$$

The derivation of equation 25 follows the same line of reasoning. We now assume that we are near the optimal amplitudes so that,

$$P\left(\boldsymbol{\Phi} \,\middle|\, D, I\right) \propto e^{-Q''/2\sigma^2}$$

where

$$Q'' = \sum_{j,k=1}^{M_{\boldsymbol{\Phi}}} \boldsymbol{\Phi}_j \boldsymbol{\Phi}_k H_{jk}$$

and H_{jk} was defined in equation 26. Using the same change of variable approach, one can show

$$\gamma_k^2 \equiv \langle \boldsymbol{\Phi}_k^2 \rangle - \langle \boldsymbol{\Phi}_k \rangle^2 = \sigma^2 \sum_{l=1}^{M_{\boldsymbol{\Phi}}} \frac{u_{lk}^2}{v_l}$$

where u_{lk} is the kth element of the lth eigenvector, v_l, of $\hat{H}$.

References:

[1] M.A.T. Figueiredo, R.D. Nowak, and S.J. Wright, "Gradient projection for sparse reconstruction: Application to compressed sensing and other inverse problems," IEEE Journal on Selected Topics in Signal Processing **1**(4), (2007).
[2] G.H. Golub, P.C. Hansen, and D.P. O'Leary, "Tikhonov regularization and total least squares," SIAM Journal on Matrix Analysis and Applications **21**(1), (1999).
[3] L.I. Rudin, S. Osher, and E. Fatemi, "Nonlinear total variation based noise removal algorithms," Physica D **60**(1–4), (1992).
[4] S. Bagchi, and S.K. Mitra, *The Nonuniform Discrete Fourier Transform and Its Applications in Signal Processing* (1999).
